# Fine Time Course Expression Analysis Identifies Cascades of Activation and Repression and Maps a Putative Regulator of Mammalian Sex Determination

**DOI:** 10.1371/journal.pgen.1003630

**Published:** 2013-07-11

**Authors:** Steven C. Munger, Anirudh Natarajan, Loren L. Looger, Uwe Ohler, Blanche Capel

**Affiliations:** 1 Department of Cell Biology, Duke University, Durham, North Carolina, United States of America; 2 Center for Genome Dynamics, The Jackson Laboratory, Bar Harbor, Maine, United States of America; 3 Program in Computational Biology and Bioinformatics, Duke University, Durham, North Carolina, United States of America; 4 Howard Hughes Medical Institute, Janelia Farm Research Campus, Ashburn, Virginia, United States of America; 5 Institute for Genome Sciences & Policy, Duke University, Durham, North Carolina, United States of America; 6 Department of Biostatistics & Bioinformatics, Duke University, Durham, North Carolina, United States of America; Seattle Children's Research Institute, United States of America

## Abstract

In vertebrates, primary sex determination refers to the decision within a bipotential organ precursor to differentiate as a testis or ovary. Bifurcation of organ fate begins between embryonic day (E) 11.0–E12.0 in mice and likely involves a dynamic transcription network that is poorly understood. To elucidate the first steps of sexual fate specification, we profiled the XX and XY gonad transcriptomes at fine granularity during this period and resolved cascades of gene activation and repression. C57BL/6J (B6) XY gonads showed a consistent ∼5-hour delay in the activation of most male pathway genes and repression of female pathway genes relative to 129S1/SvImJ, which likely explains the sensitivity of the B6 strain to male-to-female sex reversal. Using this fine time course data, we predicted novel regulatory genes underlying expression QTLs (eQTLs) mapped in a previous study. To test predictions, we developed an *in vitro* gonad primary cell assay and optimized a lentivirus-based shRNA delivery method to silence candidate genes and quantify effects on putative targets. We provide strong evidence that *Lmo4* (Lim-domain only 4) is a novel regulator of sex determination upstream of *SF1 (Nr5a1)*, *Sox9*, *Fgf9*, and *Col9a3*. This approach can be readily applied to identify regulatory interactions in other systems.

## Author Summary

The commitment of a bipotential gonad to differentiate as a testis or an ovary is governed by a dynamic transcription network that remains to be elucidated. We profiled genome-wide gene expression at <5 hr resolution during the critical one-day developmental window in XX and XY mouse gonads from the 129S1/SvImJ and C57BL/6J strains. We identified cascades of expression in both strains and show that establishment of dimorphic expression is largely due to activation and repression programs initiated by the testis pathway. Strikingly, comparison of expression differences between the two strains revealed a delay in the male program in C57BL/6J gonads, suggesting an explanation for the increased susceptibility to male-to-female sex reversal exhibited by this strain. Finally, we exploit the predictive power inherent in this temporal dataset to identify a novel candidate gene, *Lmo4*, underlying an expression QTL identified in a previous study. Confirming our prediction, knockdown of this gene in primary XY gonad cells resulted in the down-regulation of several male program genes. Our results highlight the importance of fine-scale resolution time-course measurement of expression in developmental systems to identify candidate regulatory genes and to understand general properties of the system.

## Introduction

Fate determination of the bipotential gonad results in differentiation of a testis or ovary and is crucial to the sexual differentiation of the embryo. This binary decision, known as primary sex determination, takes place at mid-gestation in the mouse. The initial pliable nature of the gonad and its rapid progress into one of two divergent, opposing fates makes it a particularly attractive model to investigate transcriptional network dynamics during fate decisions in developmental systems.

The early sexual plasticity of the mammalian gonad appears to result from a balanced, transient transcriptional network state [1]. Many genes associated later with a specific sexual fate are expressed early and at similar levels in both XX and XY gonads, a pattern indicative of lineage priming [2]. Sex determination proceeds by first establishing a bias in the transcription network toward the male (testicular) or female (ovarian) fate. In therian mammals, the Y-chromosome transcription factor, *Sry*, is the genetic trigger responsible for diverting the bipotential gonad to a testicular fate. *Sry* is expressed in XY gonads beginning at E10.5 and plays an important role in the up-regulation of *Sox9* and *Fgf9* [3]–[5]. While several genes are known to be required for adult ovarian fate [6]–[9], much less is known about the initiation of the female pathway. Subsequent to the primary fate decision in both differentiation pathways, feedback mechanisms are activated that canalize the chosen sexual fate and repress genes associated with the alternative fate. Failure to trigger or maintain one sexual fate can result in trans-differentiation to the alternative fate (i.e., sex reversal) [10]–[12].

Several lines of evidence suggest that many more important players in mammalian sex determination await discovery. First, approximately 1,500 genes are already expressed in a sexually-dimorphic pattern at E11.5, when the gonad is morphologically indistinct and still competent to sex-reverse [13]–[18]. Second, the majority of cases of human sex reversal are yet to be explained by any of the genes known to have an impact on sex determination [19].

Some inbred strains appear to be better suited than others to cope with perturbations in the sex determination pathway. For example, C57BL/6J (B6) is sensitive to XY male-to-female sex reversal in response to multiple genetic perturbations (including both Y-linked and autosomal variants), while other strains like 129S1/SvImJ (129S1) and DBA/2J (D2) are resistant to these variants (i.e., develop normal testes) [20], [21]. This differential sensitivity to sex reversal was first exploited by Eicher and colleagues in genetic studies to map regions of the B6 genome correlated with sensitivity of the XY gonad to male-to-female sex reversal [22]. More recently, using an expression QTL (eQTL) mapping approach, we identified multiple genomic regions where segregation between B6 and 129S1 markers was highly correlated with the expression levels of multiple genes of known importance to sex determination [1]. Most of these eQTL intervals harbored no genes with known roles in sex determination, and thus, likely contain novel genes in the network.

To improve our resolution of the transcriptional cascade controlling sex determination, and choose attractive candidates in eQTL intervals, we conducted a fine time course transcriptome analysis of the gonad between E11.0–E12.0, when the bipotential gonad approaches a decision point, initiates the testicular or ovarian pathway, and begins to reinforce the sexual fate decision. We profiled global gene expression at six equally-spaced intervals in XX and XY gonads from the susceptible B6 and resistant 129S1 strains, developed and trained a Hidden Markov Model (HMM) to discern the onset of sexually-dimorphic expression, and identified gene cohorts activated or repressed specifically in the testis or ovary during this brief 24-hour window of development. By comparing the onset profiles of both strains, we found that susceptibility to sex reversal in B6 XY gonads is likely due to the delayed activation of many testis pathway genes and delayed repression of many ovarian pathway genes. We exploited this detailed view of the B6 and 129S1 gonad transcriptomes to prioritize candidate regulatory genes underlying eQTLs mapped in our previous study [1]. Finally, we developed a primary cell validation assay and lentivirus-based shRNA delivery method to artificially silence *Lmo4* (Lim-domain only 4), a candidate regulatory gene within an eQTL interval. We provide strong evidence that *Lmo4* is a novel regulator of sex determination upstream of many sex-associated genes. This work provides a systematic framework for predicting and testing regulatory genes (eQTGs) underlying eQTLs that is applicable to other systems.

## Results

### A Complex, Highly Dynamic Transcription Network Underlies Gonadal Sex Determination in Mammals

**Figure 1:**
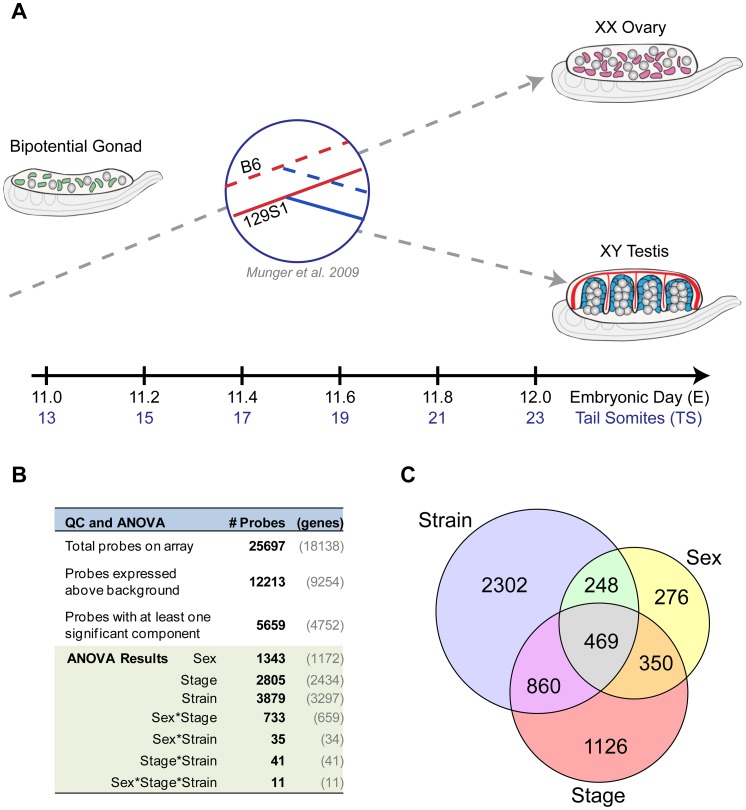
Time course analysis of the gonad transcriptome during the 24-hour period encompassing sex determination. (A) Experimental design. Total transcript abundance was profiled in XX and XY gonads at six equally spaced intervals between E11.0–E12.0 capturing the critical transition in the gonad transcriptome from a bipotential to sexually-differentiated state. The analysis was conducted in two inbred strains, C57Bl/6J (B6) and 129S1/SvImJ (129S1), with well-characterized differences in their sensitivity to sex reversal. By including XX gonads and multiple time points in the analysis, the current study expands on an earlier strain comparison of B6 and 129S1 gonads at E11.5 [1]. (B) Analysis of Variance (ANOVA) results. Nearly half of the probes on the array (n = 12,213), representing more than half of all genes (n = 9,254, shown in gray), were expressed above background levels at one or more time points between E11.0–E12.0 in XY or XX gonad samples. For a large proportion of expressed probes (n = 5,659), variation in gonad transcript abundance was significantly associated with additive effects from sex, developmental stage, and/or strain. A sex-by-stage interaction effect accounted for a significant proportion of the expression variation in 733 probes. (C) For many probes, variation in expression is driven by more than one experimental variable. A Venn diagram showing probes whose expression is affected by one or more of the additive effects of sex, stage, and strain. Values within each circle correspond to the number of probes that are significantly affected by that variable. Note that the overlaps in the Venn diagram do not capture interaction effects, but represent probes that are significantly affected by two or all three factors. For example, the 469 probes in the center region all exhibit variation in expression that can be attributed to differences in sex, stage, and strain independently, but not necessarily a sex*stage*strain effect (as occurs for 11 probes in the Anova analysis).

To elucidate the fine temporal dynamics of the gonad transcriptome during the critical fate decision to develop as a testis or ovary, we assayed total transcript abundance in XY and XX gonads at six equally spaced intervals between E11.0–E12.0 (Figure 1A). This 24-hour window captures the gonad from the time it is still bipotential to a point when it has shifted to a testis or ovarian fate. To associate variation in the transcriptome with phenotypic differences, we compared gene expression from gonads of two common inbred strains, 129S1 and B6, that differ in their susceptibility to XY sex reversal.

Total transcript abundance was measured by microarray for individual pairs of gonads for each sex/strain/stage combination (n = 74 total arrays, see Materials and Methods). A total of 9,254 genes (12,213 probes) exhibited significant expression above background in at least two replicates of one sample type, and were included in subsequent analyses (Figure 1B). Next, we fit a linear model accounting for the effects of strain, sex, stage, and two-way (e.g. sex*stage) and three-way (e.g. sex*stage*strain) interactions among these factors (Figure S1). For more than half (n = 4,752 (5,659 probes)) of the genes that passed our filtering criteria, a significant proportion of the observed variation in expression could be attributed to one or more experimental variables (Figure 1B). The individual components of sex (n = 1,172 genes/1,343 probes), stage (n = 2,434 genes/2,805 probes), and strain (n = 3,279 genes/3,879 probes), as well as the interaction effect of sex by stage (n = 659 genes/733 probes), all had significant effects on the expression of hundreds to thousands of genes. For many of these genes, expression was influenced by the additive effects of multiple components (probes in overlap regions in Venn diagram, Figure 1C). Moreover, the sex by stage interaction effect reflects the number of genes that have a sexually-dimorphic pattern of expression that changes over time in the E11.0–E12.0 window. Finally, the large number of genes with a strain effect highlights the extent to which the transcription programs vary in the B6 and 129S1 strains. These data illustrate both the complexity and dynamic nature of the transcriptional program driving sex determination during this brief but critical developmental window.

### Sexually Dimorphic Gene Expression in the Gonad Is Established by Activation and Repression Programs

**Figure 2:**
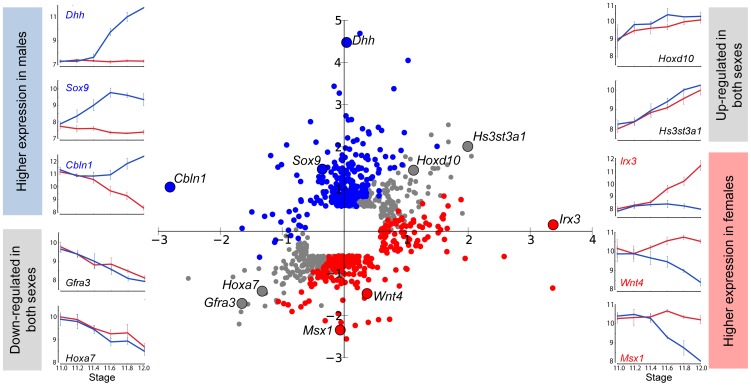
Changes in XX and XY gonads contribute to expression fold change between E11.0 and E12.0. Gene expression in XY and XX gonads was compared at the beginning and end of the 24-hour developmental window. For probes that exhibited a 1.5-fold or greater change in expression in either sex between E11.0 and E12.0, log of the Fold Change in the XY gonad is plotted on the Y-axis, and log of the Fold Change in the XX gonad is plotted on the X-axis. Probes that are similarly up-regulated or down-regulated in both sexes appear in gray in the upper right and lower left quadrants, respectively. Probes that become enriched in XY gonads relative to XX are shown in blue, while genes that become enriched in XX gonads relative to XX are shown in red. Examples from each category are highlighted, and their expression patterns in XY (blue line) and XX (red line) gonads are displayed. From this perspective, it is clear that enrichment in one sex is achieved by activation, repression, or both regulatory mechanisms.

To obtain a global view of how dimorphism is achieved during the sexual fate specification of the gonad, we calculated the fold change in both XX and XY gonads for each gene between E11.0 and E12.0 in the robust 129S1 strain. We then graphed these sex-specific fold changes for each gene on an X-Y scatter plot, with fold changes in the XY gonad appearing on the Y-axis, and changes in the XX gonad appearing on the X-axis (Figure 2, Dataset S1). Complex refractory or oscillatory patterns were not detected over this relatively short temporal window, and therefore this two-stage comparison accurately characterized overall changes in gene expression. We first note from the scatter plot that the microarrays captured the expected expression patterns of several genes with known roles in sex determination. Some of these exhibiting male enrichment (*Sox9*, *Dhh*, and *Cbln1* [23], [24]), or female enrichment (*Irx3*, *Wnt4*, and *Msx1* [25], [26]), are shown adjacent to their locations in the scatter plot. In addition to genes with sexually dimorphic expression patterns, we also identified genes that are identically-repressed or activated by both the male and female programs (genes on the diagonal in Figure 2). We hypothesize that pathways active early in both sexes are associated with a plastic bipotential state. We would expect these genes (e.g. *Gfra3* or *Hoxa7*) to be down-regulated in both sexes as sexual differentiation proceeds. In contrast, genes that are identically-activated in both sexes between E11.0–E12.0 (e.g. *Hoxd10* and *Hs3st3a1*), may be associated with transition from a sexually-primed but plastic transcriptional state to a ovarian or testicular fate regardless of the nature of the fate commitment. A subset of these genes (14, log2(fold change) >0.585 in both sexes) overlaps with the “core adrenogonadal program” previously identified in the related steroidogenic-factor-1-positive cell population [27].

This view of sexually dimorphic expression changes between E11.0 and E12.0 also revealed that higher expression in one sex can result from activation in one sex (e.g. *Dhh* in Figure 2), repression in the other sex (e.g. *Msx1*), or a combination of both mechanisms (e.g. *Wnt4*). From the scatter plot, it is evident that dimorphic expression of most genes (205 genes, log2(fold change) >0.585) expressed higher in the XY gonad occurred primarily through activation, with a small outlier group of genes (25 genes, log2(fold change) <−0.585) showing dimorphism as the result of repression in the XX gonad. Among genes showing higher expression in XX gonads, two principal gene clusters were evident: members of one cluster (77 genes) achieved dimorphism primarily through activation in the XX gonad, and members of the other (148 genes), primarily through repression in the XY gonad. This indicates that the dynamic expression changes observed during gonad fate commitment are a result of the action of activation and repression programs. We designed our following analysis to thoroughly characterize these and other aspects of the male and female transcriptional programs.

### Cohorts of Genes Can Be Resolved Based on Their Onset of Dimorphism in the Narrow Window between E11.0–E12.0

**Figure 3:**
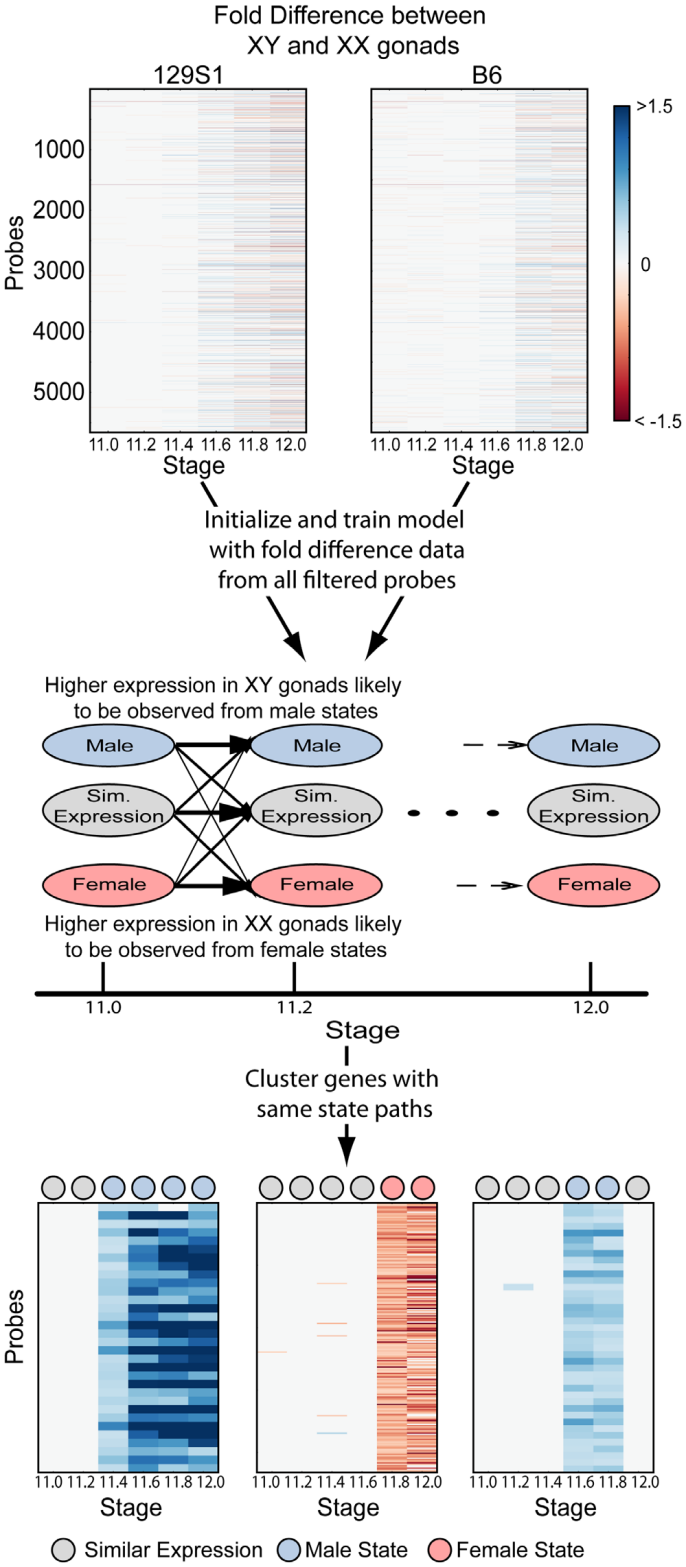
A Hidden Markov Model (HMM) to identify patterns of dimorphic expression in the gonad transcriptome. Fold differences between XY and XX gonads at each time point in both strains were calculated for all probes passing the ANOVA filtering step. This data was then used to initialize and train the Hidden Markov Model (HMM) (see Materials and Methods). The most probable (Viterbi) state path reflects possible dimorphic expression patterns between XX and XY gonads and was used to cluster genes. Heatmaps illustrate 3 clusters with state paths indicated by circles at the top of each heatmap.

To identify ordered cascades of expression and co-regulated genes, we developed a Hidden Markov Model (HMM) (Figure 3). HMMs are well-suited to the task of discerning patterns in time series data [28], [29] because they use correlations between adjacent time points to overcome noise and increase sensitivity. Briefly, the HMM was designed with 18 states, three per time point – a male (i.e. testis-enriched) state, a female (i.e. ovary-enriched) state, and a similar expression state (Figure 3, Figure S2). The fold difference of a gene's expression between XX and XY gonads at each time point was used to train the HMM. After training the model, the Viterbi state path of each gene reflected whether the gene was expressed in a sexually dimorphic fashion, the sex in which it was expressed more highly, and the times at which the gene exhibited dimorphic expression. Importantly, only 22 of a possible 729 state paths through the model were populated (Table S1, Dataset S2), indicating that despite the highly dynamic changes in the transcriptome, there are common expression trajectories by which the expression patterns can be clustered. We note that while the HMM classifies groups of genes as becoming dimorphically expressed at specific times, this is due to the discrete sampling during the window. In reality, expression changes likely occur in a continuum. Nonetheless, grouping the genes by their onset of dimorphism reveals interesting details of the regulatory programs involved.

Out of the 4,752 genes included in the analysis, 1,321 genes exhibited dimorphic expression at one or more time points between E11.0–12.0 in the 129S1 strain and similar numbers (1,037 genes) were dimorphically expressed in the B6 strain (Table S1). Interestingly, for both 129S1 and B6, once a gene established a dimorphic expression pattern, most continued in a state of sexually dimorphic expression until E12.0 (n = 1,254 genes for 129S1, n = 995 genes for B6). We refer to these genes as male- or female-enriched depending on which sex exhibited higher expression. Finally, only three genes (*Lefty2*, *Mcm6*, and *LOC233529*) in 129S1 (and none in B6) switched from being more highly expressed in one sex to the other during the duration of our window.

**Figure 4:**
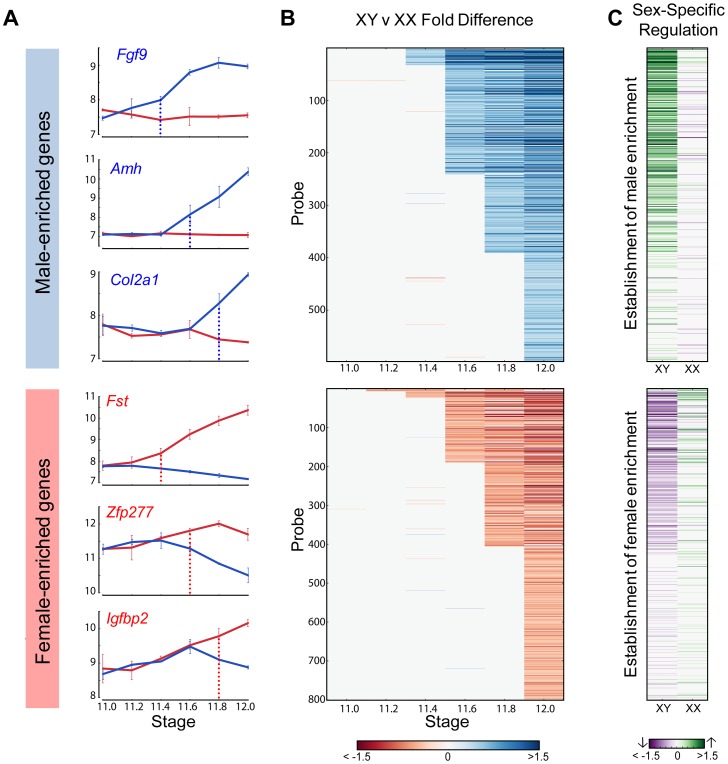
Cascades of dimorphic expression involving both activation and repression in XY gonads. (A) Examples of genes showing higher expression in XY (male-enriched genes, top panel) and XX gonads (female-enriched genes, bottom panel) from 129S1 mice. Blue and red vertical lines show the time point when dimorphic expression is significant. (B) Cascades of dimorphic gene expression identified by the HMM in XY (top panel) and XX gonads (bottom panel). Colors indicate the log fold change between XY and XX gonads at a specific time point for the 129S1 strain. The genes are arranged in order of time of onset of dimorphic expression. (C) Contribution to changes in expression between E12.0 and the time point before the onset of dimorphism are shown for each gene in (B) in XY (column 1) and XX (column 2) gonads. Top panel: male-enriched genes. Bottom panel: female-enriched genes. This analysis shows that male-enriched genes are mostly up-regulated in XY gonads while female-enriched genes are mostly down-regulated in XY gonads.

We used the HMM to cluster male- and female-enriched genes by the time of onset of dimorphic expression from E11.2 to E12.0 for the 129S1 strain (Figure 4A, B). This analysis revealed striking cascades of sexually-dimorphic male and female enrichment with the number of male- and female-enriched genes gradually increasing across time points. For example, for male-enriched genes, a single gene (*Sox9*) showed higher expression in males starting at E11.2, followed by 30 genes at E11.4, and finally 202 genes that showed sexually dimorphic expression at E12.0 (Table S1). To determine whether these cascades primarily reflected changes in one gonadal cell type or several, we compared our whole gonad data with cell type-specific gene expression data from E11.5 and E12.5 isolated XX and XY supporting cells and germ cells [2]. We found that the overlap with germ cells was low (5%) (Figure S3). In contrast, 58% of genes that became male- or female- enriched in our whole gonad transcriptome prior to E11.8 were specifically dimorphic at E11.5 or E12.5 in supporting cells. After E11.8, the overlap with the supporting cell precursors dropped to 45%. Thus, consistent with previous results, the supporting cell lineage, known to be critical for initiating the sex determination decision, is responsible for a large proportion of the sexually-dimorphic gene expression that arises in the gonad between E11.0–E12.0 [2].

To determine whether activation, repression, or a combination of both was involved in primary establishment of dimorphism for each gene, we compared gene expression in each sex before the initiation of differential expression and the E12.0 stage (Figure 4C). Note that this analysis is more sensitive than the scatter plot (Figure 2) in identifying the cause of dimorphism as it examines the trajectory of expression following the onset of dimorphism as opposed to the starting point of the analysis (E11.0).

For all genes that showed higher expression in the XY gonad by E11.8, 73% of genes (256 genes) were strongly activated (log2(fold change) >0.32, p<0.05) in XY gonads, whereas only 9.5% (34 genes) were repressed in the XX gonad (log2(fold change) <−0.32, p<0.05). In addition 5.6% (20 genes) become dimorphic through a combination of activation in the XY and repression in the XX gonad. This indicates a strong activation program in XY gonads with a much lower contribution from repression of male pathway genes in XX gonads. In striking contrast, enrichment of genes in XX gonads results not from activation in the ovary, but primarily through repression in the testis (Figure 4C, lower panel). Only 16% of probes (61 genes) that are female-enriched by E11.8 are activated in the XX gonad, while 61% (217 genes) are repressed in the XY gonad, with 7.5% (27 genes) becoming dimorphic due to a combination of activation in the XX and repression in the XY gonad. In fact, in several cases, *Msx1* for example (Figure 2), female enrichment stems exclusively from repression taking place in the testis. This indicates that in addition to the activation program, a strong repressive program is also present in the testis. While male repression of specific female pathway genes (*Wnt4*) has been known, the extent of this repressive signature is surprising.

The HMM classified *Sox9* as the earliest male-enriched autosomal gene (E11.2), reflecting its position directly downstream of *Sry* in the testis pathway [5] and affirming the fine temporal resolution of our dataset. As in previous microarray experiments using whole gonad samples [14], *Sry* was not detected above background levels in the current study. Following the up-regulation of *Sox9*, several other known crucial downstream genes such as *Fgf9*, *Amh*, and *Dhh* showed increased expression in XY gonads (Figure 4A, top panel). In the female-enriched group, *Wnt4*, one of the earliest autosomal genes known to act in ovary differentiation [6], [12], was sexually-dimorphic no earlier than E11.4. Other known female pathway genes such as *Fst* and *Axin2* became differentially expressed at the same stage or immediately following the dimorphic expression of *Wnt4*. All the male-enriched genes that were activated prior to *Sox9* and female-enriched genes enriched prior to *Wnt4* are Y- and X-linked genes, respectively (Figure S4 and S5). Particularly interesting are the 7 X-linked genes that exhibited higher expression in XX gonads starting at E11.2 (Figure S5). The cell-type specific data indicate that these genes are all highly expressed in germ cells and likely reflect the reactivation of the inactive X chromosome in XX germ cells at this stage [30].

### Activation of the Male and Repression of the Female Differentiation Pathways in the XY Gonad Are Delayed in the Sensitive B6 Strain

After characterizing the temporal dynamics of XY and XX gonad transcriptomes from the 129S1 strain that is resistant to XY sex reversal, we determined how the transcriptome varied in B6, a strain that is sensitive to XY sex reversal in response to multiple genetic perturbations [20], [31], [32]. While previous studies showed that male-enriched genes were expressed at a higher level and female-enriched genes at a lower level in 129S1 compared to B6 E11.5 XY gonads [1], it was not clear whether this strain difference was a result of the difference in expression levels, time of onset, or a combination of both. To address these questions, we profiled global gene expression in XY and XX gonads from B6 at the same six time points spanning the critical 24-hour window (E11.0–E12.0).

**Figure 5:**
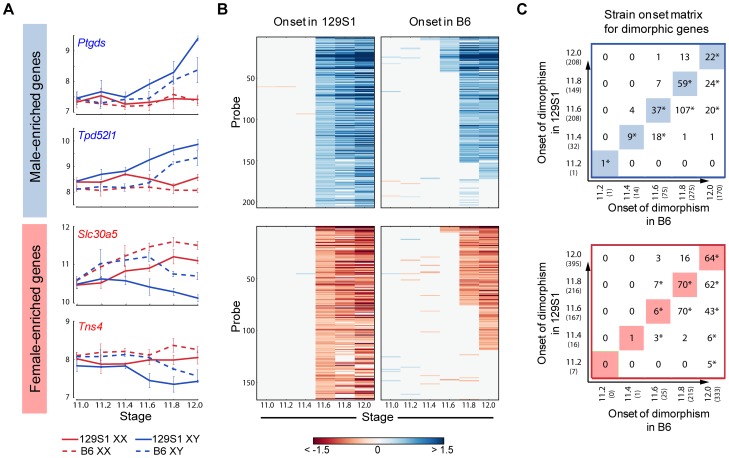
Dimorphic expression of multiple male- and female-enriched genes in B6 is delayed compared to 129S1 mice. (A) Expression of male- (top panel) and female-enriched (bottom panel) genes. Dimorphic expression for these genes is delayed by ∼5 hours in B6 compared to 129S1. (B) Heatmap showing dimorphic expression at E11.6 in 129S1 and comparison of same genes in B6. While a few genes show earlier dimorphic expression in B6 mice compared to 129S1, the dominant pattern shows a ∼5 hr delay between B6 and 129S1 mice. (C) Matrix showing the time of onset of dimorphism in 129S1 and B6 mice for male-enriched (top panel) and female-enriched (bottom panel) genes. For example, out of the 32 male-enriched probes that became dimorphically expressed at E11.4 in 129S1, 9 probes were also dimorphically expressed starting at E11.4 in B6 while 18 showed dimorphic expression starting at E11.6 in B6 mice. However, 4 genes are dimorphic in B6 XY gonads at E11.4, but not in 129S1 until E11.6. * indicates significant overlap with p<0.001 evaluated by a hypergeometric test. The highlighted diagonals show the number of genes showing similar onset of dimorphism in 129S1 and B6 mice. Note that some genes that are male- or female-enriched in one strain do not show dimorphism in the other strain.

We used the HMM to identify male- and female-enriched genes in B6 mice (Figure S6). In good agreement with the data from 129S1 mice, these genes showed activation and repressive programs in XY gonads, and were strongly biased toward dimorphic expression in supporting cells. We then compared the timing of onset of sexually dimorphic gene expression between 129S1 and B6 (Figure 5). We observed a clear, consistent temporal shift in the onset of sexually-dimorphic expression in many genes in the susceptible B6 strain (Figure 5A, B). Specifically, for male-enriched genes, a comparison of strain onset distribution profiles revealed a statistically significant ∼5-hour delay in B6 relative to 129S1 (Figure 5C, upper panel). For example, 208 probes became enriched in 129S1 XY gonads relative to XX starting at E11.6 (Figure 5B). When the same probes were examined in the sensitive B6 strain, only 37 became dimorphic at the same stage, while a majority (n = 107) became male-enriched ∼5 hours later at E11.8. Another 20 from this set did not become dimorphic in B6 until E12.0, and 35 probes that were male-enriched in 129S1 at E11.6 failed to become sexually-dimorphic in B6 by E12.0. Even in the case of genes that became male-enriched at the same time point in B6 and 129S1, a comparison of XY v. XX fold difference at the onset of dimorphism indicated that a majority of these genes (65.6%) show a higher male v. female fold difference in 129S1 than in B6 (Figure S7A, Binomial test p-value<0.005). This difference may reflect a more robust activation mechanism driving the male differentiation pathway in 129S1, or it could reflect a delay in expression onset in B6 that is less than ∼5 hours and therefore smaller than the minimum resolution threshold of this analysis.

Importantly, this delayed onset pattern of male-activated genes in B6 does not appear to stem from a difference at the top of the cascade in *Sry* expression level during this critical window. Although *Sry* was not detected above background in these arrays, there was no significant difference in expression levels between 129S1 and B6 between E11.2–E12.0 by qRT-PCR (Figure S8A). It should be pointed out that the high variability in *Sry* abundance observed among individual pairs of XY gonads could mask a small but real strain effect for *Sry* expression levels. In contrast, *Sox9* is more robustly up-regulated in 129S1 relative to B6 (Figure S7B), an observation we confirmed by qRT-PCR (Figure S8B). By E11.8 expression of *Sox9* in B6 XY gonads has caught up with expression in 129S1. Some of the delay in onset of male-enriched genes at later stages in B6 may be due to this initial deficiency in the robustness of *Sox9* activation.

Note that the delayed onset pattern was not observed for every gene that became male-enriched over this 24-hour period. For example, of the 208 probes that were male-enriched starting at E11.6 in 129S1, a few (*Gstm2*, *Etv5*, *Gas7*, and *Mybphl*) became sexually-dimorphic *earlier* in B6. Similarly, of the 75 genes that showed male-enrichment in B6 at E11.6, 11% (n = 8, including S*chip1*, *Lpl*, *Socs2*) become male-enriched later in 129S1 or not at all before E12.0. This indicates that although much of the male differentiation pathway is delayed in B6, this pattern is unlikely to be due to a more general delay in gonad differentiation.

Female-enriched genes exhibited a similar significant ∼5 hour delay in B6. However, this strain delay stems from the later repression of female genes in XY gonads (Figure 5B lower panel). As a consequence, B6 XY gonads are exposed to higher levels of female pathway genes for a longer period relative to 129S1 XY gonads. For example, of the 166 probes that become female-enriched in 129S1 starting at E11.6, only six exhibit a similar expression pattern in B6, while 70 become female-enriched ∼5 hours later, another 43 probes become dimorphic at E12.0, and 47 fail to reach a sexually-dimorphic state by E12.0 (Figure 5C, lower panel). Similar to the male-enriched genes, 62.4% of female-enriched genes that become dimorphic at the same time in both strains show a higher fold difference in 129S1 than in B6 (Figure S7C, p<0.005).

In summary, our evidence argues against a general developmental delay in B6 and suggests that the increased sensitivity of B6 XY gonads to sex reversal stems from a delay in the activation of male pathway genes downstream of *Sox9*, combined with a consequent delay in the repression of female pathway genes.

### Strain Differences in Temporal Gene Expression Can Predict Candidate Genes that Underlie Expression Quantitative Trait Loci

In addition to providing a more comprehensive view of the global transcription dynamics driving male and female sex determination in the robust 129S1 and sensitive B6 strain backgrounds, this fine temporal expression data provided a means for narrowing eQTLs to identify novel regulators of sex determination. In previously published work, we mapped 19 regions of the genome in an F2 intercross population where genetic variation between B6 and 129S1 was correlated with differences in gene expression for one or more genes associated with sex determination [1]. Eight of these regions were correlated with the expression of multiple genes, yet none of these prominent “trans-band eQTLs” harbored an obvious candidate gene with a known role in the sex determination process. Unfortunately, most of the eQTL regions identified in this initial coarse mapping were too large to functionally test every gene in the interval.

We established filtering criteria based on temporal strain expression and genomic data to prioritize candidate genes within the eight trans-band eQTLs (Table 1). Briefly, protein-coding genes in the interval were considered as candidates if they were expressed at one or more time points between E11.0–E12.0 in XY samples (eQTLs were mapped only in XY samples; therefore, the causative gene underlying an eQTL should be expressed in the XY gonad). Based on this list, we analyzed each candidate within the interval for strain differences in expression levels or time of onset, and prioritized genes with strain-dimorphic patterns. We investigated whether each gene harbored one or more polymorphisms (SNPs, insertion/deletions) that differed between B6 and 129S1 and might affect its expression or function. Only those genes with characterized variation within 10 kb up- and down-stream of the transcription start site (TSS) were prioritized for further analysis. Finally, we interrogated the Mammalian Phenotype (MP) browser to identify any genes in the region with a characterized knockout phenotype affecting sex determination (MP:0002210, abnormal sex determination), or a known relationship with any of the target genes it was predicted to regulate. We tailored our candidate search strategy to each individual eQTL, and exploited prior information about the expression or function of the target genes for that region. Thus, we expected that genes involved in regulating early gonadogenesis genes would be expressed in both sexes at an early stage, whereas those regulating the male or female pathway would be more likely to exhibit sexually dimorphic gene expression.

**Table 1:** Identification of candidate genes in prominent trans-band eQTLs based on dynamic expression patterns.

eQTL Chr: interval (MGI Genes)	Controlled Transcripts	Expression pattern/Function of transcripts	Expressed in XX/XY gonad	Sexually Dimorphic	Strain Dimorphic	Abnormal SD phenotype (MP:0002210)	Best Candidate(s)
**1:** 33–49cM (364)	*Sry, Sox9, Fgf9, Ptgds, Cbln1*	Male pathway	97	11	32	4	*Serpine2, Inha, Myl1, Igfbp5, Efhd1*
**3:** 65–76cM (60)	*Fog2 (Zfpm2), Ctnnb1, Pld1, SF1 (Nr5a1), Dapk1, Dock4, Cbln1, Rec8L1 (Rec8), Fgf9, Rspo1, Col9a3, Smpdl3b, Gata4, Socs2, Wt1, Asns*	Early gonadogenesis, Female pathway, Male pathway	20	6	13	0	***Lmo4*** *, Gbp1/2/3, Ccbl2, Bcl10*
**5:** 26–46cM (276)	*Sphk1, Mmd2, Trim47, Dhh, Tpd52l1, Serpine2*	Male-enriched	61	12	15	5	*Pdgfra, Kit, Ppargc1a, Igfbp7*
**12:** 33–49cM (219)	*Rpgrip1, Wt1, Dock4, Fog2 (Zfpm2), Axin2, Pld1, Pdgfd, Ctnnb1, Dax1 (Nr0b1)*	Early gonadogenesis, Female pathway	72	9	17	3	*Gtf2a1, Hspa2, Mlh3*
**14:** 31–39cM (174)	*Cbln1, Serpine2, Gng13*	Male-enriched	58	5	23	6	*Gata4, Ptk2b, Piwil2*
**15:** 36–58cM (526)	*SF1 (Nr5a1), Cst9, Pglyrp1, Rec8L1 (Rec8), Dtna, Mmd2, Centb1 (Acap1), Tpd52l1, Defb19*	Early gonadogenesis, Female-enriched, Male-enriched	208	29	77	13	*Sp1, Dhh, Amhr2, Sbf1, Smc1b, Mov10l1, Pfdn5, Pick1*
**17:** 19–39cM (367)	*Taf7l, Defb19, Ren1, Slco3a1, Smoc2, Pglyrp1*	Male-enriched, Female-enriched	123	11	37	5	*Txndc2, Dazl, Rab31*
**19:** 36–41cM (108)	*Fst, Slco3a1, Smpdl3b*	Female-enriched, Male-enriched	49	6	22	1	*Tmem180, Sema4g, Pax2*

Strain differences in temporal gene expression are informative for predicting regulatory genes underlying trans-band eQTLs. Multiple criteria were established to identify the best candidate genes in the trans-band eQTLs mapped in Munger et al. [1]. First, the putative regulatory gene must be expressed above background at or before E11.5 to exert any effects on downstream target genes. Genes implicated in the list of target genes that exhibited a sexually-dimorphic expression pattern consistent with the sexual differentiation pathway (male or female) were prioritized. Genes that met both of the above criteria, and exhibited strain expression differences (either in overall levels of transcript abundance or in timing of onset of sexual dimorphism) in a pattern consistent with the observed allelic effects for that eQTL, were prioritized as the highest candidates. A few eQTLs harbor genes in which abnormal sex determination phenotypes have been noted in the null mutant mouse, and these genes were given similar high priority. Using these criteria, confidence intervals containing 60–526 protein coding genes were narrowed down to eight or fewer high priority candidates.

**Figure 6:**
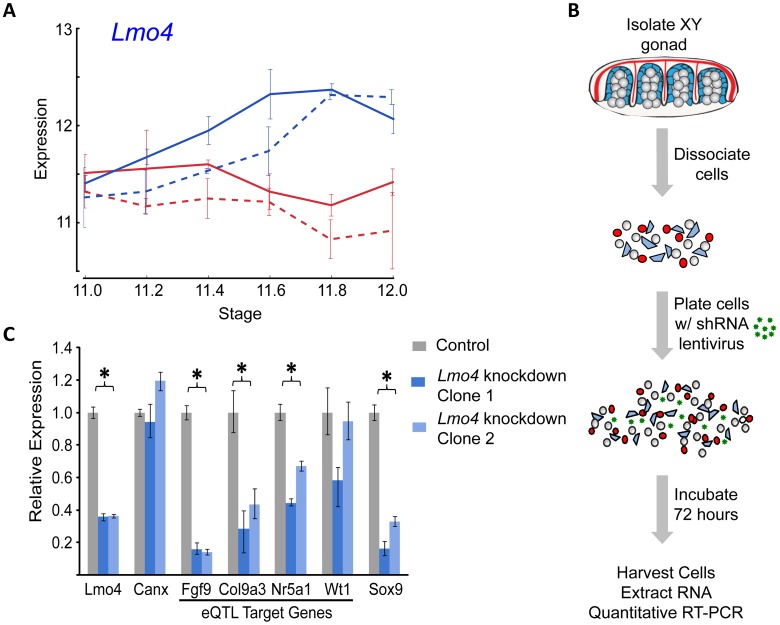
Validation of *Lmo4* as a novel regulator of gene expression in the fetal gonad. (A) *Lmo4* exhibits an expression pattern indicative of a role in sex determination and consistent with expectations for a gene underlying a strain eQTL regulating early sex determination and male pathway genes. It is expressed at similar levels in XY and XX gonads before E11.6, becomes enriched in XY gonads as early as E11.6 in both strains, and shows reduced expression levels in B6 (dashed lines), consistent with the observed allelic effects of the Chromosome 3 eQTL. (B) E12.5 XY gonads were dissected free of the mesonephroi, pooled by sex, dissociated into single cell suspensions, plated on tissue culture plates at t = 0 with lentiviral particles containing shRNA targeted to the candidate gene of interest, and cultured for 72 hours. Quantitative RT-PCR was conducted to assay expression of predicted targets. (C) Lentiviral shRNA-mediated knockdown of Lmo4 in cultured XY primary gonadal cells resulted in the consistent down-regulation of multiple Chromosome 3 eQTL target genes relative to the nontargeting control (gray bar) using two different shRNA hairpins targeting Lmo4 (light/medium blue bars in graph). Expression was normalized to the housekeeping gene *Gapdh*. Both male pathway genes, *Fgf9* and *Col9a3*, were significantly down-regulated following Lmo4 knockdown with both clones. Similarly, one of the putative targets with a role in early gonadogenesis, *SF1/Nr5a1*, was significantly reduced, however expression of the other gene involved in early gonadogenesis, *Wt1*, was not significantly affected by *Lmo4* knockdown. The important male pathway regulator *Sox9* was found to be significantly down-regulated as a result of *Lmo4* knockdown. *Canx* (a second normalization gene not predicted to be a target of LMO4) showed no difference in expression compared to the control. Error bars show minimum and maximum expression. Significance was calculated by comparing control and data across all independent runs.

In total, for the eight prominent trans-band eQTLs mapped in our previous study [1], of which the average interval contains ∼300 genes (range = 60–526 genes), we narrowed each down to, at most, eight promising candidates. Of particular interest, the distal Chr 3 region was strongly associated with the expression of nearly one-third of all the genes in our previous mapping study [1], including known regulators of early gonadogenesis (*Fog2/Zfpm2*, *SF1*/*Nr5a1*, *Gata4*, *Wt1*, and *Ctnnb1*) and both the female (*Ctnnb1*, *Rspo1*) and male (*Fgf9*) differentiation pathways. A total of six genes were identified as candidates based on their strain-dimorphic expression patterns, but only the transcription cofactor *Lmo4* (lim domain only 4) exhibited a dynamic pattern consistent with a role early in both pathways and an additional male-specific role downstream of the sexual fate decision. *Lmo4* is expressed at similarly high levels in both sexes until E11.4 (Figure 6A), and becomes male-enriched as early as E11.6. Importantly, while *Lmo4* was up-regulated in B6 XY gonads at the same time as in 129S1, there was a significant strain effect with expression in 129S1 being higher. This observation is consistent with the observed allelic effect for the eQTL (B6129SF2 gonads that were homozygous for the 129S1 allele exhibited higher expression of target genes). Finally, there is a significant amount of genetic variation in *Lmo4* between 129S1 and B6, including an insertion in the 3′ UTR in 129S1 as well as multiple intronic SNPs and indels [33]. Based on these selection criteria, we elected to focus on developing a functional assay for *Lmo4*.

### Validation of Lmo4 as the Causative Gene Underlying the Trans-band eQTL on Distal Chromosome 3

Historically, moving from a list of candidate genes to a validated quantitative trait gene (QTG) has represented the largest hurdle (in both resources and time) to success in complex trait mapping studies in the mouse. To address this problem, we optimized a lentivirus-mediated shRNA delivery method to artificially silence candidate regulatory genes with high efficiency in dissociated gonad primary cells from E12.5 XY gonads (Figure 6B). As a positive control, we utilized pre-designed and validated shRNA clones (Sigma MISSION) packaged in lentiviral vectors to silence *Sox9* expression in primary gonadal cell culture, and quantified the expression of known downstream targets (Figure S9). Lentiviral-mediated knockdown resulted in a nearly 80% reduction in *Sox9* expression relative to a nontargeting control sample. Two of the three known targets (direct or indirect) of SOX9, *Amh* [34] and *Fgf9* [4], were down-regulated significantly following *Sox9* knockdown. *Ptgds*, the third known target of SOX9 [35], is not expressed at high levels in cultured gonad primary cells, and we could not detect a change in *Ptgds* expression following *Sox9* knockdown. However, a marker of the female pathway, *Fst* [36], showed a significant and greater than 2-fold up-regulation in this assay (p<0.016). Thus, this *in vitro* assay recapitulates well-characterized genetic interactions that occur in the gonad *in vivo*.

We extended our analysis to test *Lmo4* as a candidate regulator underlying the Chr 3 eQTL. We silenced *Lmo4* expression to 36% of a nontargeted control (p<0.001) (Figure 6C). Although the degree of knockdown was relatively modest, it was observed consistently in three independent trials and with two shRNA clones. Importantly, silencing *Lmo4* expression by two-thirds resulted in the consistent, significant down-regulation (p<0.05) of three of the four putative eQTL targets measured by qRT-PCR. *Fgf9*, *Col9a3*, and *SF1/Nr5a1* were significantly down-regulated by both shRNAs (Figure 6C). Down-regulation of a fourth target of the Chr 3 eQTL, *Wt1*, was not statistically significant (p<0.13). Interestingly, although it was not identified as a Chr 3 eQTL target in our original mapping study, *Sox9* expression is significantly down-regulated following *Lmo4* knockdown by both shRNA clones (p<0.001). Note that the predicted targets (*Fgf9*, *Col9a3*, and *SF1/Nr5a1*) of the Chr 3 eQTL region are those that are affected by the different alleles in B6 and 129S1. Even though *Sox9* did not map as a target of the Chr 3 eQTL in our study, it might not be differentially regulated by *Lmo4* between B6 and 129S1, yet could still be a target of *Lmo4*. In total, these experiments provide strong support for *Lmo4* as the transcriptional regulator underlying the Chr 3 trans-band eQTL and may reveal additional regulatory interactions that were undetected in the eQTL mapping study.

## Discussion

The transcriptional cascades that control development of multicellular organisms are a central focus in modern biology [37]. It is now evident that transcriptional regulation involves the coordinated action of a cohort of players including transcription factors, chromatin remodelers, non-coding RNAs, and epigenetic modifications. An important step in identifying the specific players in this network and deconvolving their effects on the transcriptional program is a detailed characterization of the transcriptome during the developmental process. Our efforts in this paper were focused on the critical 24-hour window when the gonad begins to transition from a bipotential primordium to a testis or ovarian fate. To that end, we sampled global transcript abundance at 3× finer granularity than previous studies, and in the process discovered multiple temporal cohorts of sexually-dimorphic genes in this brief window. Importantly, we found that sexually-dimorphic gene expression patterns during this period are primarily driven by activation and repression cascades in the XY gonad. Most male-enriched genes are activated in the XY gonad but remain unexpressed or unchanged in the XX gonad. In contrast, female–enriched genes acquire that pattern mostly by a combination of repression in the XY gonad and continued activation in the XX gonad. A ∼5-hour delay in both the activation of the testis pathway and repression of the ovarian pathway likely underlies the sensitivity of the B6 strain to XY sex reversal. We applied this new temporal expression resource to prioritize eQTL intervals mapped in our previous study. Finally, we developed a primary cell-based RNAi assay, and used it to validate a candidate new regulator of sex determination.

### Gonadal Sex Determination Is Orchestrated by a Highly Dynamic Transcriptome

Previous microarray studies profiled transcript abundance in whole gonads or isolated cell populations at two or more time points before and after the sex determination decision [2], [13]–[15]. These datasets served as important resources for the field. However, the temporal resolution around the critical stage of sex determination was limited in all but one study to 24-hour intervals (Nef sampled at E10.5, E11.0, and E11.5). It was evident from these earlier studies that the gonad transcriptome changed very little between E10.5–E11.0, that the difference between E11.0–E11.5 was significant, and between E11.5–E12.5 the testis and ovarian transcriptomes are highly sexually dimorphic. We predicted that information about the sequential order of gene activation/repression during the E11.0–E12.0 window would be valuable. Using data from the fine time course, we designed an HMM to precisely separate genes based on their position in the transcriptional cascade. As opposed to other clustering methods such as k-means clustering [38], HMMs are able to both account for the time dependence in the data and exploit this layer of information to identify patterns often obscured by noise prevalent in microarray data. We note that this HMM can be readily extended to time course expression analyses in other systems.

Our analysis identified waves of sexually-dimorphic gene expression in the 24-hour window following the onset of *Sry* expression, which suggest regulatory cascades. We note that while the HMM identifies dimorphically expressed genes as being dimorphically expressed at distinct time points, this is a result of the sampling times of our transcriptome analysis. Finer sampling in this window is likely to reveal that genes grouped together at a time point show minor differences in timing of the onset of dimorphism. Previous work indicated that the supporting cell lineage is the first lineage in the gonad to show sexually dimorphic expression followed by other gonadal cell lineages after E11.5 [2]. Consistent with this, over half (58%) of the genes we identified that became sexually dimorphic prior to E11.8 could be specifically assigned to the supporting cell lineage prior to E11.8 with 5% showing dimorphism in germ cells. The discrepancies in the overlap are likely due to the increased sensitivity of the HMM to identify dimorphically expressed genes and the conservative measure of dimorphic expression used in the cell-type specific expression study.

As expected, we observed a strong signature of gene activation associated with up-regulation of the testis pathway in XY gonads. However, we were surprised by the extent of the repressive program that silences female pathway-associated genes in the XY gonad following activation of the male pathway. Testing of candidates from our study will be an important step towards identifying the factors responsible for these patterns of expression in the male and female program. Based on their early onset of sexually-dimorphic expression, several genes are promising candidates to play an early regulatory role in the male and female pathways (Table S2, Text S1). *Sox13* is a member of the SOX protein family that lacks an activation domain but can repress Wnt signaling by forming a complex with the β-catenin cofactor, TCF1 [39], [40]. *Mef2c* is activated in the XY gonad at E11.6 and has been shown to interact with *Sox9* in chondrocytes [41]. Among genes that showed female-enrichment, *Gtf2a1*, a general transcription factor that is part of the initiation complex for PolII recruitment [42], became dimorphic at E11.4 in the XX supporting cell lineage and *Tcea3*, a known PolII elongation factor became dimorphic at E11.6. Interestingly, female-enriched TFs such as *Zfp277*, *Runx1*, *Lef1*, *Lhx9* and *Msx1* were strongly down-regulated in XY gonads. Conversely, *Irx3* showed strong activation in XX gonads, and has been predicted to have a function during ovarian differentiation independent of *Foxl2* and *Wnt4* [43].

### Sensitivity to Sex Reversal in B6 Stems from the Delayed Onset of the Male Pathway Downstream of Sox9

Strain differences in resistance to sex reversal upon perturbation of the sex determination network have been the focus of several studies. The importance of the timing of the antagonistic testis and ovarian programs to B6-associated sex reversal was first proposed by Eicher in 1983 [44], based on the finding that introduction of a *Mus domesticus* Y chromosome (Y^Dom^, or Y^POS^) onto a B6 genetic background led to sex reversal [20]. Sex reversal in this case was later shown to be associated with the delayed onset of *Sry* [45]. However, this work did not explain why the B6 strain is more susceptible to sex reversal in cases where a weak allele of *Sry* is not involved [37].

Here we showed that the onset of sexually dimorphic gene expression was delayed by approximately five hours in the “unperturbed” (i.e. wildtype) B6 strain compared to 129S1. This delay is consistent across the cascade starting from E11.4 with genes that are both up- and down-regulated in XY gonads. Interestingly, we detected no significant difference by qRT-PCR in the level of *Sry* expression between the strains; however we cannot rule out a difference in the onset of *Sry* expression prior to the window of our analysis. Nonetheless, *Sox9* is up-regulated at the same stage (E11.2) in B6 and 129S1. Despite this agreement, the activation of many downstream genes in the male pathway is delayed in B6. In our previous microarray comparison of B6 and 129S1 testes at E11.5 [1], *Sox9* was found to be enriched in B6 relative to 129S1 XY gonads at E11.5, in contrast to the current study, where *Sox9* levels are lower in B6 until E11.8–E12.0 (Figure S7B). This discrepancy may stem from small developmental staging differences in the pooled gonads used in the previous study, as *Sox9* levels are changing very rapidly at E11.5. However, as this and other recent studies [32], [46] illustrate, the system may be sensitive to minor fluctuations in gene expression between E11.0–E11.5. Thus, the slightly lower level of *Sox9* expression that we detected in B6 relative to 129S1 XY gonads might contribute to the delayed onset timing of downstream genes in B6. In addition, our fine time course data here helps explain the previously observed higher expression of female-enriched genes in B6 compared to 129S1. Specifically, the observed difference at E11.5 is a consequence of the delayed repression of female-enriched genes in B6 XY gonads.

### Prediction and Validation of Lmo4 as a Novel Regulator of Sex Determination

Previous transcriptome and genetic mapping studies produced gene lists or large intervals with candidate regulators of sex determination [1], [13]–[16], [18], [22], [47]. The bottleneck in applying the results of these studies has been the inability to prioritize between several dozen candidates and then test these candidates in a manner that is inexpensive and efficient. We have addressed both these deficiencies in the current study. We used our fine time course dataset in conjunction with a previous eQTL study and cell-type expression data to identify candidate regulators of sex determination.

To overcome the hurdle of testing candidate regulators, we developed an RNAi assay to silence the expression of candidate genes, and then monitored the expression of putative downstream target genes after knockdown. As predicted, shRNA-mediated silencing of the transcription cofactor *Lmo4* resulted in the down-regulation of known important regulators of early gonadogenesis (*SF1/Nr5a1*) and the male pathway (*Sox9* and *Fgf9*). This provides strong evidence that in addition to previously characterized roles during development in the neural tube [48]–[50], neural crest [51], cortex [52], and thymus [53], *Lmo4* is also a regulator of sex determination in the gonad. However, this does not preclude the possibility that other genes on distal Chr 3 have roles during sex determination and control the expression of one or more of the 16 eQTL target genes. To point, four of the other candidate regulators identified in this region (*Gbp1/2/3*, and *Ccbl2*) are expressed at similar high levels in both sexes before E11.4, and then become down-regulated specifically in XY gonads at or after E11.6. This pattern predicts a role for these genes in the female differentiation pathway. Future assays to overexpress these candidates in XY primary cells or silence them in XX primary cells will assess their potential as regulators for one or more of the Chr 3 eQTL target genes.

In closing, our fine temporal analysis of the gonad transcriptome revealed multiple cascades of sexually-dimorphic gene activation and repression during the critical first 24 hours of sex determination. This information provides a valuable resource for future experiments to identify novel genes, pathways, and network motifs associated with sex determination in particular, but also organ differentiation in general. We replicated this analysis in a strain that exhibits a unique sensitivity to sex reversal, and showed that the compromised capacity to buffer genetic perturbation in B6 is most likely due to a consistent ∼5-hour delay in the activation of a large portion of the male pathway and subsequent down-regulation of much of the female pathway. We integrated the temporal strain expression data with genetic mapping data that identified regions associated with gene expression in the gonad at E11.5, and in so doing were able to narrow down large intervals to a small set of the best candidate genes. Finally, we optimized lentivirus-mediated RNAi knockdown in cultured gonad primary cells, and used this assay to validate *Lmo4* as a novel sex determination gene. Importantly, this validation strategy is easily scalable, and we expect that this assay will be a valuable first step to test potential regulators and in assembling a transcriptional network of sex determination.

## Materials and Methods

### Ethics

All animals were maintained and experiments were conducted according to the Institutional Animal Care and Use Committee of the Duke University Medical Center and NIH guidelines (Permit Number: A168-11-07).

### Mice, Dissection, Developmental Staging, and Genotyping

For the time course microarray study, C57BL/6J (stock no. 000664) and 129S1/SvImJ (stock no. 002448) mice were obtained from The Jackson Laboratory. CD-1 outbred mice were used (strain code 022, Charles River) in the gonad primary cell assays.

Timed matings were established for B6 and 129S1, and embryos were collected from dams between embryonic day (E) 11.0–12.0. Embryos were individually staged by counting tail somites (ts) distal to the hindlimbs: E11.0, E11.2, E11.4, E11.6, E11.8, and E12.0 corresponds to 13, 15, 17, 19, 21, and 23 ts, respectively [1], [54]. For each strain at each time point, three individual pairs of XX and XY gonads from at least two separate litters were collected. The chromosomal sex of each embryo was determined by PCR on head DNA using primers to detect *Kdm5c/Kdm5d* (5′-TGAAGCTTTTGGCTTTGAG-3′ and 5′-CCGCTGCCAAATTCTTTGG-3′). Gonads were dissected away from mesonephroi in sterile PBS (Gibco/Invitrogen, cat no. 1490-144) and stored in RNAlater RNA stabilization solution (Ambion, cat no. AM7024) at −20C until all samples were collected. To minimize contamination and RNA degradation, all surgical instruments and surfaces were treated with RNaseZAP RNase decontamination fluid (Ambion, cat no. AM9780), followed by 70% EtOH in DEPC-treated water, before and during the dissection procedure.

### Microarray Processing - RNA Isolation, Labeling, and Hybridization

For the microarray analyses, at least three biological replicate samples were profiled for each strain/stage/sex (n = 74 total arrays), with one exception (n = 2 replicates for 129S1 E12.0 XY). Total RNA was first extracted from individual pairs of E11.0–E12.0 XX and XY gonads (separated from mesonephroi) with the RNeasy Micro kit with on-column DNase digestion (QIAGEN, cat no. 74004) following the manufacturer's protocol. Total RNA was eluted in 14 ul RNase-free water (not DEPC- treated), and 2 ul were used to quantify RNA concentration on a NanoDrop ND-2000 (Thermo Scientific). Only samples with >100 ng of total RNA and an A280∶A260 ratio of >1.6 were included in the expression analyses.

From each total RNA sample, mRNA was selectively reverse transcribed with oligo(dT) primers to T7-labelled cDNA, and then amplified by in vitro transcription (IVT) to produce biotinylated cRNA using the Illumina TotalPrep Amplification Kit (Ambion/Life Technologies, cat no. AMIL1791) according to manufacturer's instructions. cRNA concentration was quantitated on the NanoDrop ND-2000, and as necessary, individual samples were concentrated in a vacuum centrifuge. 750 ng of biotinylated cRNA (in ∼10 ul volume) were hybridized to Illumina MouseRef-8 v2.0 BeadChips (Illumina, cat no. BD-202-0202) according to Illumina protocols, and array intensity was measured on an iScan scanner (Illumina). To minimize potential for batch effects to confound analysis, individual samples were assigned to 8-sample BeadChips using a balanced design.

### Microarray Data Processing and Temporal Coexpression Analysis

Microarray data files were imported into GenomeStudio software (Illumina, V2010.1), and raw expression values for each sample extracted. Expression values were quantile normalized and log2 transformed using the R package Beadarray [55]. Probes that had a detection p<0.005 in at least two replicates for any sample type were used for analysis. Data are publicly accessible in GEO (accession number GSE41948).

### ANOVA Analysis

The ANOVA analysis was conducted using the R package Limma [56]. A sex by strain by stage factorial analysis was conducted as outlined in [57]. The model included the sex, strain, and stage variables, the sex*strain, sex*stage and strain*stage two-way interaction terms, and a three-way interaction term sex*strain*stage. The model was fit for all the probes that had reliable expression (detection p<0.005) in at least two replicates of any one sample using the lmFit function in the Limma package. The statistical significance of each of the terms was evaluated using the eBayes function in Limma. Probes that did not have a significant difference (Benjamini-Hochberg adjusted p<0.05) for at least one of the variables were excluded from further analysis.

### HMM to Identify Dimorphic Expression

Hidden Markov Models (HMMs) are generative probabilistic models that explicitly model the observed data as being emitted by a ‘hidden’ biological state (here, male or female enrichment). Further, transition probabilities between states capture the time dependencies in data between adjacent time points. Inference algorithms allow for computing the most probable state paths that give rise to the observed data, and accounts for noise inherent in observed data. The modeling of time dependencies between biological states, and accounting for noisy observations, makes HMMs particularly well suited to analyze time course microarray data [28], [29].

We designed a left-to-right HMM with three states per time point (Figure 3). The three states correspond to male state (with higher expression in males), female state (with higher expression in females) and similar expression state (with no difference in expression between the two sexes). The observed data on which the model was trained and clustered was the quantized Fold Difference (FD) of the log2 normalized values between XX and XY gonads at each time point. Note that a fold change of expression of 1.25 corresponds to an FD of 0.3219, fold change of 1.5 to an FD of 0.585 and a fold change of 2 to an FD of 1. Limma was used to calculate FD between XX and XY gonads at each time point for each strain. If a specific comparison did not have a p-value<0.05, or |FD|>0.3219 then the FD for that comparison was set to 0. The FD was then quantized into symbols as follows:

Symbol s - Similar expression in XX and XY gonads [−0.3219 < FD < 0.3219].

Symbol *m_1_* - Higher expression in XY gonads [0.3219 < FD < 0.5850].

Symbol *m_2_* - Higher expression in XY gonads [0.5850 < FD < 1].

Symbol *m_3_* - Higher expression in XY gonads [1 < FD]

Symbol *f_1_* - Higher expression in XX gonads [0.3219 > FD > −0.5850].

Symbol *f_2_* - Higher expression in XX gonads [0.5850 > FD > −1].

Symbol *f_3_* - Higher expression in XX gonads [1 > FD].

The symbols *m_1_*, *m_2_*, *m_3_*, and *f_1_*, *f_2_*, *f_3_* indicate varying levels of confidence in the differential expression between XX and XY gonads.

For each gene, for each strain there were 6 symbols indicating the FD between XX and XY gonads across the time window. For example, for *Sox9* in the 129S1 strain, the following FDs were observed at the 6 time points – 0, 0.77, 1.41, 2.41, 2.30, and 1.97. Following the rules listed above, this was quantized as s, *m_2_*, *m_3_*, *m_3_*, *m_3_*, *m_3_*.

The emission probabilities of the HMM were initialized as shown in Figure S2B to reflect the intuitive meaning of the states and the possible observed symbols from each state. Note that all probabilities were initialized as being non-zero. After training was completed, emission probabilities still reflected the intuitive meaning of the states (Figure S2B).

The transition probabilities between states were initialized as follows (Figure S2A). Observed symbols were first classified into male, female and similarly expressed – symbols *m_1_*, *m_2_*, *m_3_* into state *M*, symbols *f_1_*, *f_2_*, *f_3_* into state *F*, symbol *s* to state *S*. Transitions between all combinations of states in adjacent time points were counted and normalized to make transition probabilities from each node sum to 1. A pseudocount of 1 was added to all possible transitions (transition between states in adjacent time points) to initialize all probabilities as non-zero.

The HMM was trained using the Baum-Welch algorithm for 200 iterations with data from both strains for all the probes that passed the filtering criteria and were shown to have a significant effect for at least one variable in the ANOVA analysis. The state path for the observed FDs for each of the probes was computed using the Viterbi algorithm. Probes with the same state paths were clustered together.

### shRNA Clones and Lentivirus Production

Pre-validated gene-specific MISSION shRNA clones (Sigma Aldrich; *Sox9* pLKO.1 clones: TRCN0000086165, TRCN0000086167; *Lmo4* pLKO.1 clones: TRCN0000084373, TRCN0000084375; Nontargeting Controls – TurboGFP shRNA SHC004, eGFP shRNA SHC005) and lentiviral packaging and envelope plasmids (Addgene; pCMV-dR8.2 dvpr ID# 8455, pMD2.G ID# 12259) were purchased as bacterial stocks, and high quality plasmid DNA was isolated from overnight liquid LB cultures with a Maxiprep kit (QIAGEN, cat no. 12162) following manufacturer's instructions and quantitated on a NanoDrop ND-2000.

Lentivirus production followed the Addgene 4-day protocol with slight modifications (www.addgene.org/tools/protocols/pLKO/) [58]. All work with lentiviruses was performed in a BSL2+ hood following approved biosafety procedures. On Day 1, for each sample, 5×10^6^ HEK-293T/17 cells (ATCC cat no. CRL-11268) were suspended in 10 ml of Dulbecco's Modified Eagle Medium (DMEM, Gibco cat no. 11995) +10% Fetal Bovine Serum (FBS) without antibiotics, plated to 10 cm cell culture plates, and incubated at 37°C, 5% CO_2_ overnight. Late in the afternoon of Day 2, 10 ug of pLKO.1 shRNA plasmid, 7.5 ug of pCMV-dR8.74 dvpr packaging plasmid, and 2.5 ug of pMD2.G envelope plasmid DNA were suspended in Opti-mem serum-free medium with 60 ul X-tremeGENE HP DNA transfection reagent (Roche, cat no. 06 366 236 001) in a 3∶1 ratio to a total volume of 600 ul, incubated at 25°C for 20 minutes, then applied drop-wise to the 10 cm plate containing HEK-293T/17 cells at 60–80% confluency, swirled gently to disperse evenly but not dislodge cells from the plate, and incubated at 37°C, 5% CO_2_ overnight (12–18 hours). On day 3, media containing the transfection reagent was removed carefully and decontaminated in >10% bleach. Next, 5.5 ml of fresh viral growth medium (vGM, containing Neurobasal medium (Gibco, cat no. 21103-049) supplemented with 10% FBS, 0.5 mM L-glutamine (Gibco, cat no. 25030-149), and 1× Antibiotic-Antimycotic (Gibco cat no. 15240-062)) was added carefully to the side of the plate so as not to disturb the transfected virus-producing cells, and incubated at 37°C, 5% CO_2_ overnight. Late in the afternoon of day 4, the virus-containing vGM was harvested with a 10 ml syringe, and filtered through a 0.45 um PES syringe filter (Whatman, cat no. 6780-2504) into sterile 2.0 ml polypropylene cryo-vials. Viral media was stored at 4°C for use within 5 days, or at −80°C for long-term storage. All laboratory materials that came into contact with viral particles were treated as biohazardous waste and autoclaved according to BSL2+ safety practices.

### Gonad Primary Cell Assays

The effect of silencing candidate regulatory genes was assayed in dissociated gonad primary cell cultures. Timed matings were established for CD-1 mice, and embryos were collected from dams at E12.5. Gonads were dissected away from the attached mesonephroi, sexed by visual inspection for testis cords, and XY gonads from a litter were counted and pooled. Pooled XY gonads were then dissociated in 0.25% Trypsin-EDTA (1×, Gibco cat no. 25200-056) for 15 minutes at 37°C with slight agitation, followed by centrifugation at 4000 rpm for 5 minutes, washed once with DMEM (Gibco cat no. 11965) followed by centrifugation, and suspended in Opti-Mem (Gibco cat no. 11058-021) supplemented with 1% FBS. Cells from one pair of XY gonads were determined to be sufficient for one well of a 24-well culture plate, and the amount of suspension liquid was calculated by multiplying 250 ul by the number of pairs of XY gonads in the pooled sample.

Following the dissociation and wash steps, 250 ul gonad primary cells were immediately added to individual wells of a 24-well cell culture plate, and 250 ul of the appropriate lentivirus-containing vGM was added to each well in a BSL2+ hood. In addition to wells designated to assay target gene shRNA-mediated knockdown, separate wells containing XY gonad primary cells from the same litter were infected with the non-targeting eGFP shRNA (SHC005) and/or TurboGFP shRNA (SHC004) controls. Plates were incubated at 37°C 5% CO_2_ for 68–72 hours and cell viability was monitored daily with a light microscope. Virus production could be monitored visually for the TurboGFP control infected cells using a fluorescence microscope.

### qRT-PCR

Following 68–72 hours incubation, total RNA was isolated from shRNA lentivirus-infected gonad primary cells using Trizol reagent (Life Technologies, cat no. 15596-018). Briefly, lentivirus-containing culture media was first removed from each well and disposed in bleach. Next, 400 ul of Trizol was added to the adherent cells in each well, allowed to sit at room temperature for 3–5 minutes, after which the lysate was transferred to 1.5 ml microcentrifuge tubes. Subsequent RNA isolation steps follow Munger et al. [1]. Total RNA was quantified on a NanoDrop ND-2000, treated with DNaseI (Life Technologies, cat no. 18068-015), and converted to cDNA using the iScript cDNA synthesis kit (Bio-Rad, cat no. 170-8891) following manufacturer's instructions.

Gene expression was quantified by quantitative RT-PCR (qRT-PCR) on a StepOnePlus Real-time PCR System (Life Technologies). For qRT-PCR, each analysis was performed in technical triplicate in a total volume of 20 ul reaction mix containing 2 ul cDNA template, 4 ul 1 uM gene-specific forward and reverse primers, 10 ul 2× Quantace SensiMix SYBR (Bioline, cat no. QT615-02), and 4 ul RNase-free water. The list of qRT-PCR primers can be found in Table S3; most have been previously published [1], [12]. All primer sets were tested for efficiency and found to work optimally with the ΔC_t_ method [59]. Within a sample, target gene C_t_ thresholds value were determined and normalized to *Gapdh*. Differences between target gene shRNA and non-targeting control shRNA samples were compared using the ΔΔC_t_ method as described previously [59]. Significance of expression differences between samples was assessed using a t-test.

## Supporting Information

Dataset S1 **Scatter plot gene lists.** This excel file contains the genes and their fold changes that were used to construct the scatter plot in Figure 2. The fold changes are in log2 scale and are for each sex between the initial (E11.0) and final (E12.0) time points. Fold differences were computed using limma.
(XLSX)

Dataset S2 **HMM state paths for each probe included in HMM analysis.** This table contains the genes included in the HMM analysis and their state path through the trained HMM for both strains of mice. Each state path consists of 6 symbols, referring to in order their state at the 6 time points between E11.0 and E12.0. A 0 state refers to similar expression, M to male-enriched expression, and F to female-enriched expression. For example, genes expressed higher in males starting at E11.2 and continuing through the end of the window will have the state path 0MMMMM.
(XLSX)

Figure S1 **Examples showing significant difference in expression for each of the variables in the ANOVA analysis.** (A) Sex effect (*Ddx3y*): XY gonads show higher expression than XX gonads. (B) Stage effect (*Ltbp4*): Expression is higher at later time points across strains and sex. (C) Strain effect (*Myo6*): 129S1 mice show higher expression regardless of sex and stage. (D) Sex-by-stage effect (*Gstm1*): XY gonads show higher expression starting at E11.6. (E) Sex-by-strain effect (*Xist*): B6 XX gonads show higher expression compared to 129S1 XX gonads. (F) Stage-by-strain effect (*Ptpro*): B6 gonads show higher expression at later stages. (G) Sex-by-stage-strain (*Hapln3*): 129S1 XY gonads starting at E11.8 show significantly different expression compared to all other sample types.
(JPG)

Figure S2 **State transition and emission probabilities of the Hidden Markov Model (HMM).** (A) State transition probabilities for the HMM (Fig. 3) after (before) training with the Baum-Welch algorithm. Numbers in the E11.0 column show probability of a gene's expression starting in the male, female or similar expression state after (before) training. Transition probabilities are shown for each pair of transitions from one time point to the next for E11.2–E12.0. Colors of the cells indicate the state at that time point. First three rows in each column show transition from a male state at the previous time point, the middle three show transition from a similar expression state and the last three rows show transition from a female state at the previous time point. For example, after training, the probability of transitioning from a similar expression state at E11.6 to a male expression state at E11.8 is 0.06. (B) State emission probabilities for the three states before (left panels) and after training (right panels). Emission probabilities for discretized fold changes were initialized by hand. After training, emission probabilities still reflect the intuitive meaning of the states. For example, higher expression in XY gonads is likely to be observed in emissions from the male state. Emission probabilities for the states were tied across time points.
(JPG)

Figure S3 **Genes male- or female-enriched in whole gonads between E11.0 and E12.0 are primarily dimorphic in the supporting cell lineage of the gonad in 129S1 mice.** The cascade of genes that become dimorphically expressed between E11.2 and E12.0 (same data as in Figure 4) was cross-referenced with cell-type specific expression datasets analyzed at E11.5 and E12.5 [2]. Column 1 shows overlap with genes expressed dimorphically in supporting cells while column 2 shows overlap with genes expressed dimorphically in germ cells. Rows are colored blue or red where the probe was dimorphically expressed and higher in XY cells or higher in XX cells, respectively. The highest overlap is seen with the supporting cells for both male- and female-enriched genes.
(JPG)

Figure S4 **X-linked and Y-linked genes that are dimorphically expressed across the E11.0 – E12.0 window.** (A) Expression of *Ddx3y*, *Eif2s3y*, and *Jarid1d* in 129S1 gonads (blue – expression in XY gonads, red – expression in XX gonads). All three genes are Y-linked and are expressed higher in XY gonads across the E11.0 – E12.0 window. (B) Expression of *Xist*, *Utx*, and *Eif2s3x* in 129S1 gonads. All three genes are X-linked and are expressed higher in XX gonads across the E11.0 – E12.0 window.
(JPG)

Figure S5 **X-linked genes showing higher expression starting at E11.2 are germ-cell enriched.** (A–G, left column) 7 genes showing higher expression in XX gonads in 129S1 mice, likely due to the higher number of germ cells in 129S1 mice [S15]. (A–G, right column) Corresponding expression in male (solid line) and female (broken line) in germ cells (green) and supporting cells (purple) from cell-type specific expression data. Note that *4932441K18Rik* expression was not captured in the cell-type specific expression dataset. All genes show enriched expression in germ cells.
(JPG)

Figure S6 **Detailed characterization of dimorphic expression in B6 gonads reveals properties similar to 129S1 gonads.** (A) Examples of genes showing higher expression in XY (male-enriched genes, top panel) and XX gonads (female-enriched genes, bottom panel) from B6 mice. Blue and red vertical lines show the time of onset of dimorphic expression. (B) Cascades of dimorphic gene expression identified by the HMM in XY (top panel) and XX gonads (bottom panel). Colors indicate the fold difference between B6 XY and XX gonads at a specific time point. The genes are arranged in order of increasing time of onset of dimorphic expression. (C) Contribution to changes in expression between E12.0 and the time point before the onset of dimorphism are shown for each gene in (B) in XY (column 1) and XX (column 2) gonads. Top panel: male-enriched genes. Bottom panel: female-enriched genes. This analysis shows that male-enriched genes are mostly up-regulated in XY gonads while female-enriched genes are mostly down-regulated in XY gonads. (D) The cascade of genes dimorphically expressed was cross-referenced with cell-type specific expression datasets analyzed at E11.5 and E12.5 [2]. Column 1 shows overlap with genes expressed dimorphically in supporting cells while column 2 shows overlap with genes expressed dimorphically in germ cells. Rows are colored blue or red where the probe was dimorphically expressed and higher in XY cells or higher in XX cells, respectively. As with 129S1 gonads, the highest overlap is seen with the supporting cells for both male- and female-enriched genes in B6 gonads.
(JPG)

Figure S7 **Robust onset of dimorphism in 129S1 mice compared to B6 mice.** (A, C) Scatterplot showing XY vs. XX fold difference (A) and XX vs. XY fold difference (C) at the onset of dimorphism for male- and female-enriched genes that are activated at the same stage. Fold difference between 129S1 XY and XX gonads at the onset of dimorphism are plotted on the y-axis and the fold difference between B6 XY and XX gonads at the onset of dimorphism on the x-axis. Onset of dimorphism is more robust in the 129S1 strain for both male- and female-enriched genes. (B) *Sox9* becomes dimorphic at E11.2 in 129S1 and B6 gonads. However, the fold difference between XY and XX gonads is higher at E11.2 in 129S1 mice.
(JPG)

Figure S8 **129S1 and B6 XY gonads show no significant difference in** ***Sry*** **expression but a small difference in** ***Sox9*** **expression as assayed by qRT-PCR.** (A) *Sry* expression levels are similar in 129S1 and B6 XY gonads between E11.2–E12.0. No statistically significant (p<0.1) differences are detected at any time point in this analysis, however high variability among individuals may mask a small but biologically meaningful strain effect for *Sry* transcript abundance in this window. (B) *Sox9* expression shows significantly different expression (p<0.1 (*) and p<0.05 (**)) at the 17 and 18 tail somite stage.
(JPG)

Figure S9 **Lentiviral mediated knockdown of** ***Sox9*** **in gonad primary cell culture results in down-regulation of male-enriched genes.** Knockdown of *Sox9* resulted in down-regulation of known male-enriched genes such as *Amh* and *Fgf9* and up-regulation of female-enriched gene *Fst*. However, *Ptgds*, a known male-enriched gene [35], does not show down-regulation.
(JPG)

Table S1 **Numbers of genes (probes) in each Viterbi state path identified by the HMM for both 129S1 and B6 mice.** Each state path has six states, one for each time point, and genes having the same state path are clustered together. Only 3 genes switch from showing higher expression in one sex to higher expression in the other. Most genes that become dimorphic continue showing dimorphism throughout the E11.0–E12.0 window.
(JPG)

Table S2 **Transcription Factors (TFs) and cofactors that are either male- or female- enriched genes and show dimorphic expression by E11.6 in supporting cells in 129S1 mice.** Genes showing delayed onset of dimorphism in B6 are shown in grey boxes. Known interactions of the TFs that are relevant to sex determination are also shown.
(JPG)

Table S3 **List of primers used for qRT-PCR.**
(JPG)

Text S1 **List of references from Supplemental Material.**
(DOCX)
