# Supplementary figures and images for: Fine Time Course Expression Analysis Identifies Cascades of Activation and Repression and Maps a Putative Regulator of Mammalian Sex Determination
Source: PLoS Genet. 2013 Jul 11;9(7):e1003630. doi: 10.1371/journal.pgen.1003630 (PMC3708841; doi:10.1371/journal.pgen.1003630)

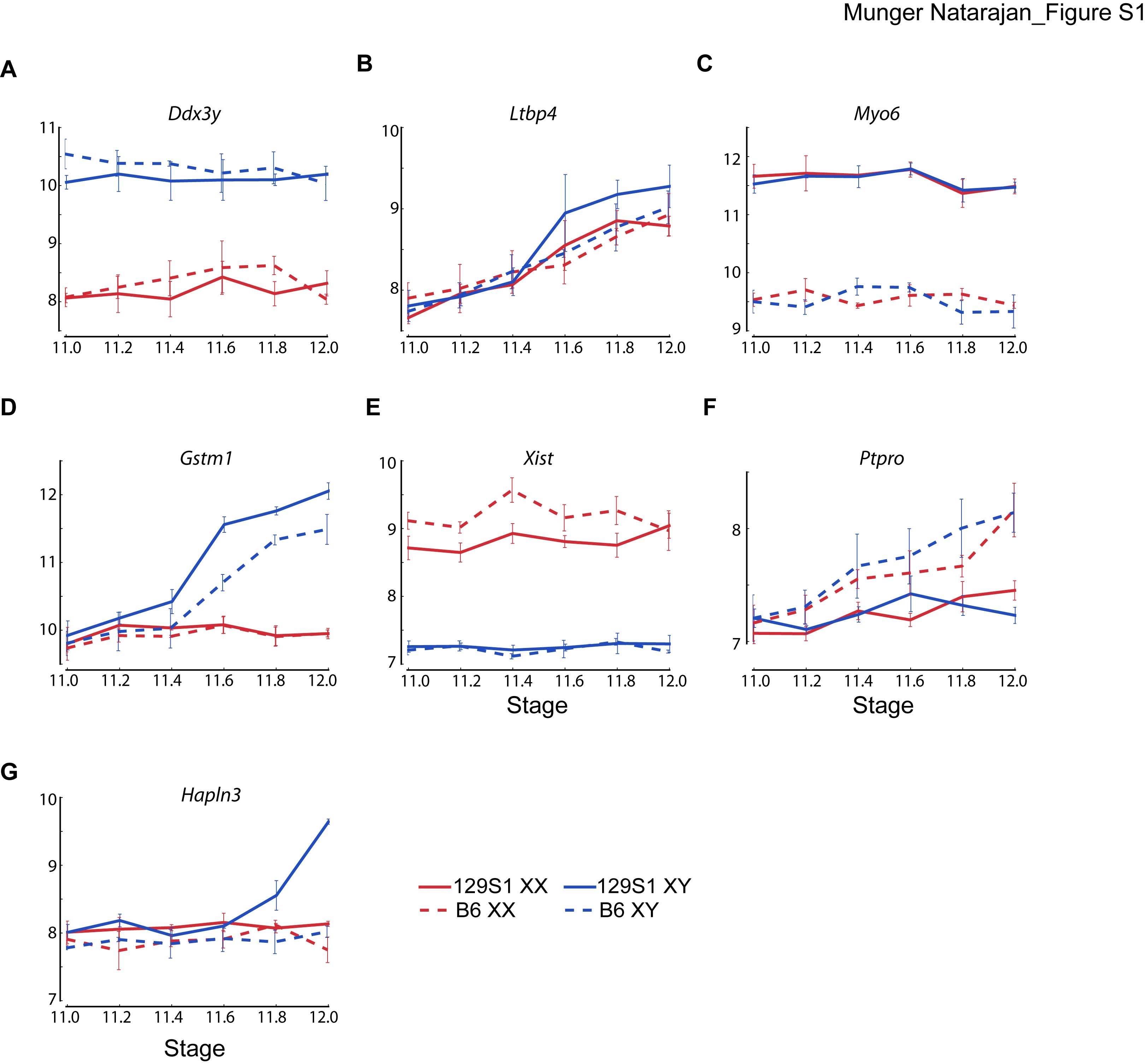

Supplement: Figure S1 — Examples showing significant difference in expression for each of the variables in the ANOVA analysis. (A) Sex effect (Ddx3y): XY gonads show higher expression than XX gonads. (B) Stage effect (Ltbp4): Expression is higher at later time points across strains and sex. (C) Strain effect (Myo6): 129S1 mice show higher expression regardless of sex and stage. (D) Sex-by-stage effect (Gstm1): XY gonads show higher expression starting at E11.6. (E) Sex-by-strain effect (Xist): B6 XX gonads show higher expression compared to 129S1 XX gonads. (F) Stage-by-strain effect (Ptpro): B6 gonads show higher expression at later stages. (G) Sex-by-stage-strain (Hapln3): 129S1 XY gonads starting at E11.8 show significantly different expression compared to all other sample types. (JPG) [file pgen.1003630.s003.jpg]

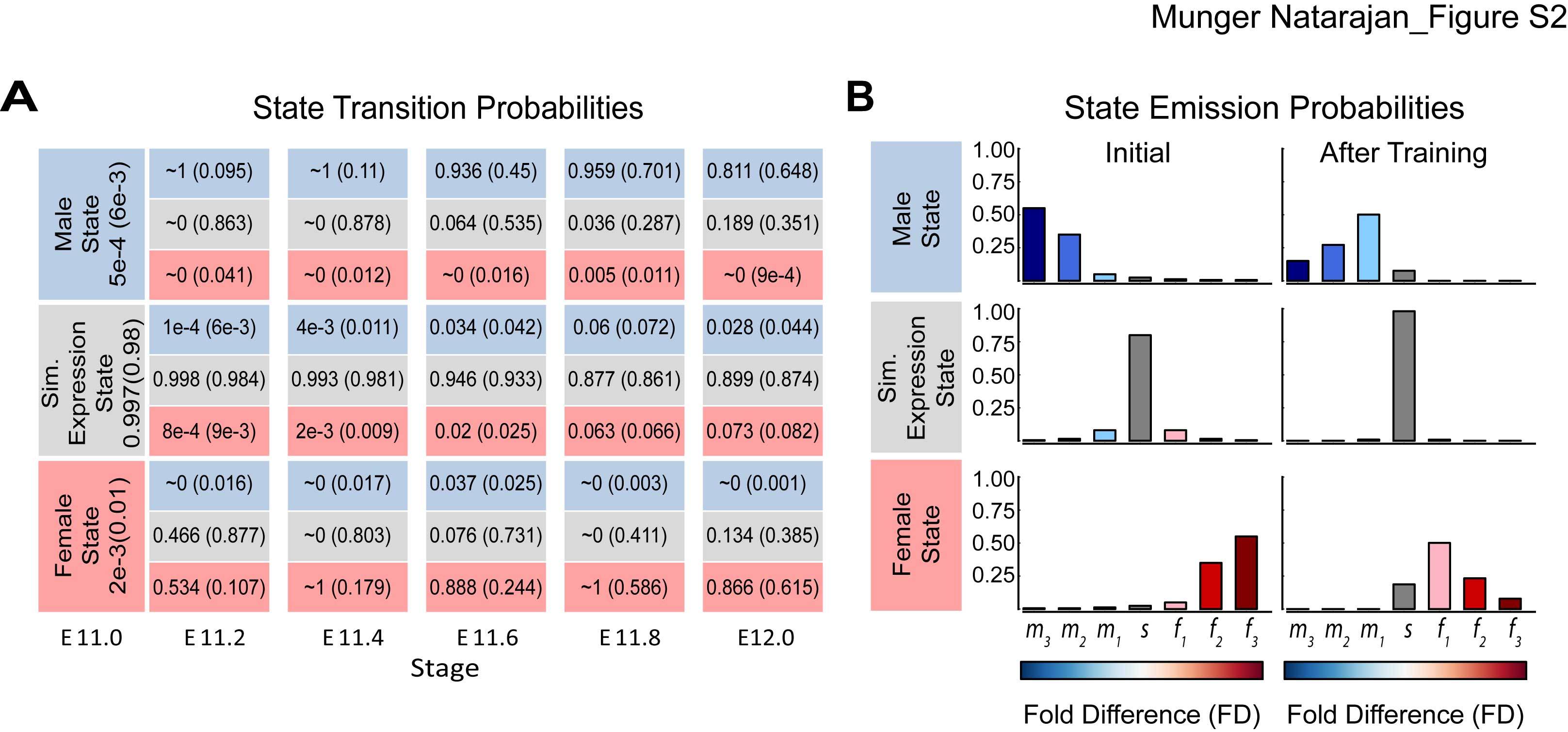

Supplement: Figure S2 — State transition and emission probabilities of the Hidden Markov Model (HMM). (A) State transition probabilities for the HMM (Fig. 3) after (before) training with the Baum-Welch algorithm. Numbers in the E11.0 column show probability of a gene's expression starting in the male, female or similar expression state after (before) training. Transition probabilities are shown for each pair of transitions from one time point to the next for E11.2–E12.0. Colors of the cells indicate the state at that time point. First three rows in each column show transition from a male state at the previous time point, the middle three show transition from a similar expression state and the last three rows show transition from a female state at the previous time point. For example, after training, the probability of transitioning from a similar expression state at E11.6 to a male expression state at E11.8 is 0.06. (B) State emission probabilities for the three states before (left panels) and after training (right panels). Emission probabilities for discretized fold changes were initialized by hand. After training, emission probabilities still reflect the intuitive meaning of the states. For example, higher expression in XY gonads is likely to be observed in emissions from the male state. Emission probabilities for the states were tied across time points. (JPG) [file pgen.1003630.s004.jpg]

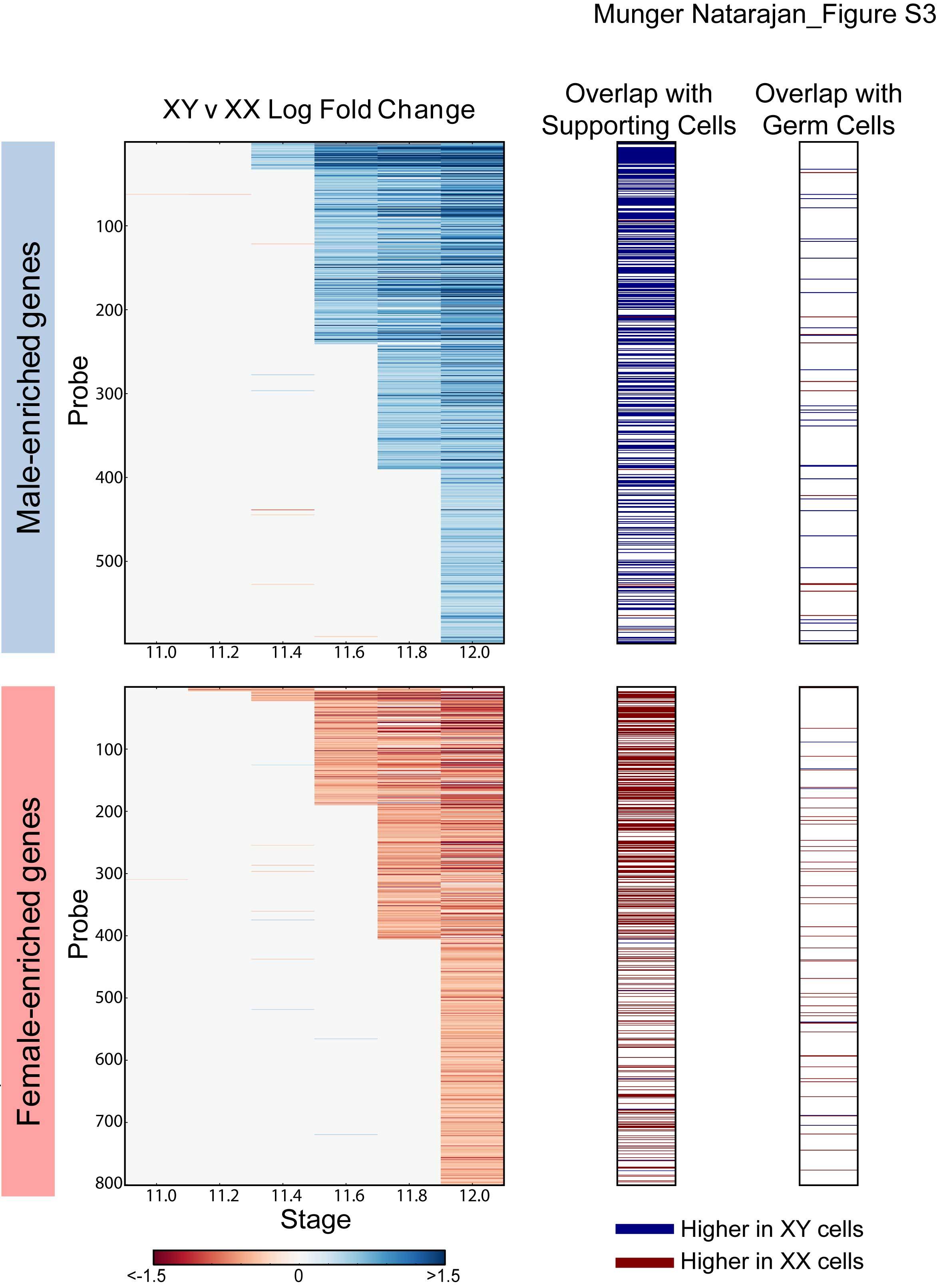

Supplement: Figure S3 — Genes male- or female-enriched in whole gonads between E11.0 and E12.0 are primarily dimorphic in the supporting cell lineage of the gonad in 129S1 mice. The cascade of genes that become dimorphically expressed between E11.2 and E12.0 (same data as in Figure 4) was cross-referenced with cell-type specific expression datasets analyzed at E11.5 and E12.5 [2]. Column 1 shows overlap with genes expressed dimorphically in supporting cells while column 2 shows overlap with genes expressed dimorphically in germ cells. Rows are colored blue or red where the probe was dimorphically expressed and higher in XY cells or higher in XX cells, respectively. The highest overlap is seen with the supporting cells for both male- and female-enriched genes. (JPG) [file pgen.1003630.s005.jpg]

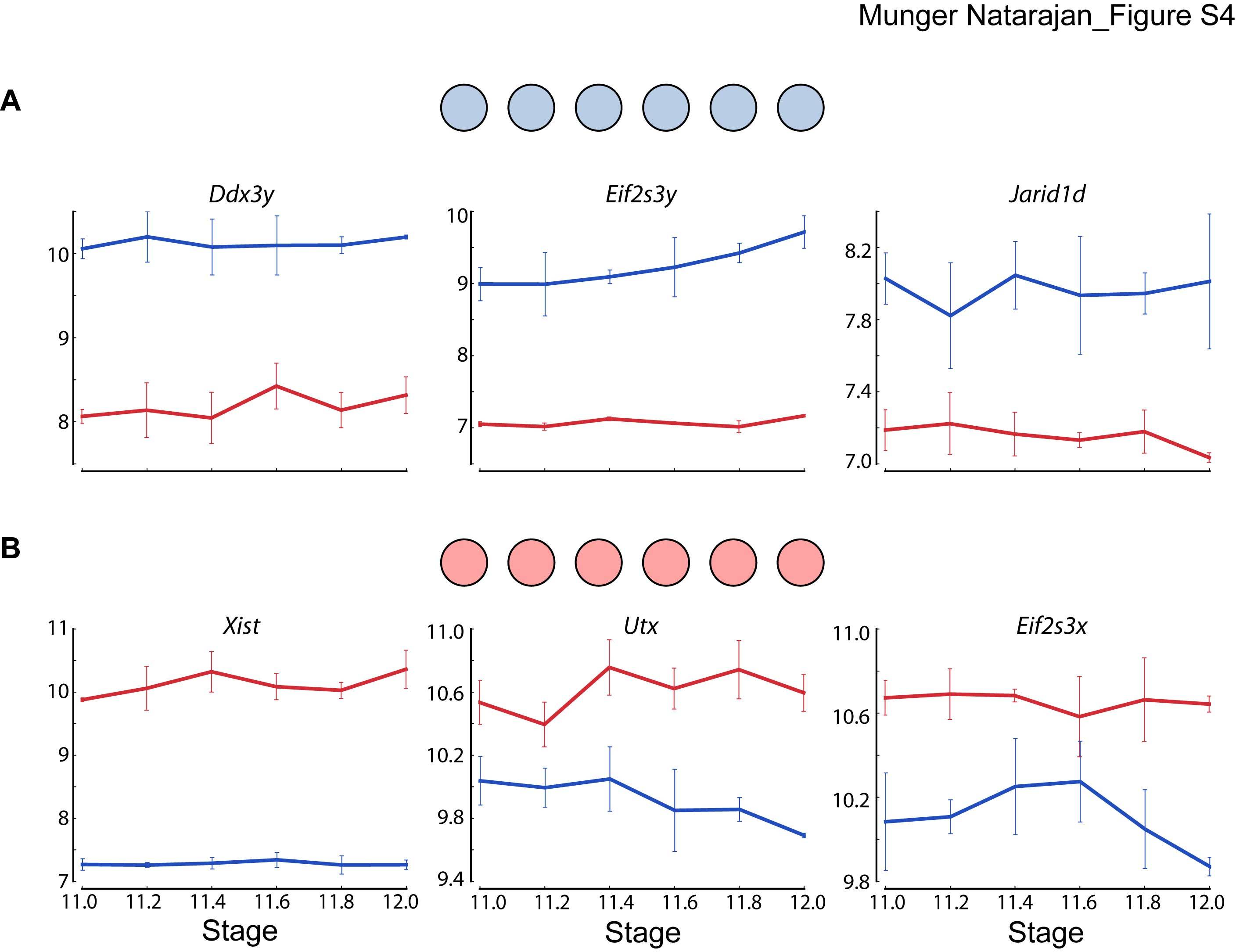

Supplement: Figure S4 — X-linked and Y-linked genes that are dimorphically expressed across the E11.0 – E12.0 window. (A) Expression of Ddx3y, Eif2s3y, and Jarid1d in 129S1 gonads (blue – expression in XY gonads, red – expression in XX gonads). All three genes are Y-linked and are expressed higher in XY gonads across the E11.0 – E12.0 window. (B) Expression of Xist, Utx, and Eif2s3x in 129S1 gonads. All three genes are X-linked and are expressed higher in XX gonads across the E11.0 – E12.0 window. (JPG) [file pgen.1003630.s006.jpg]

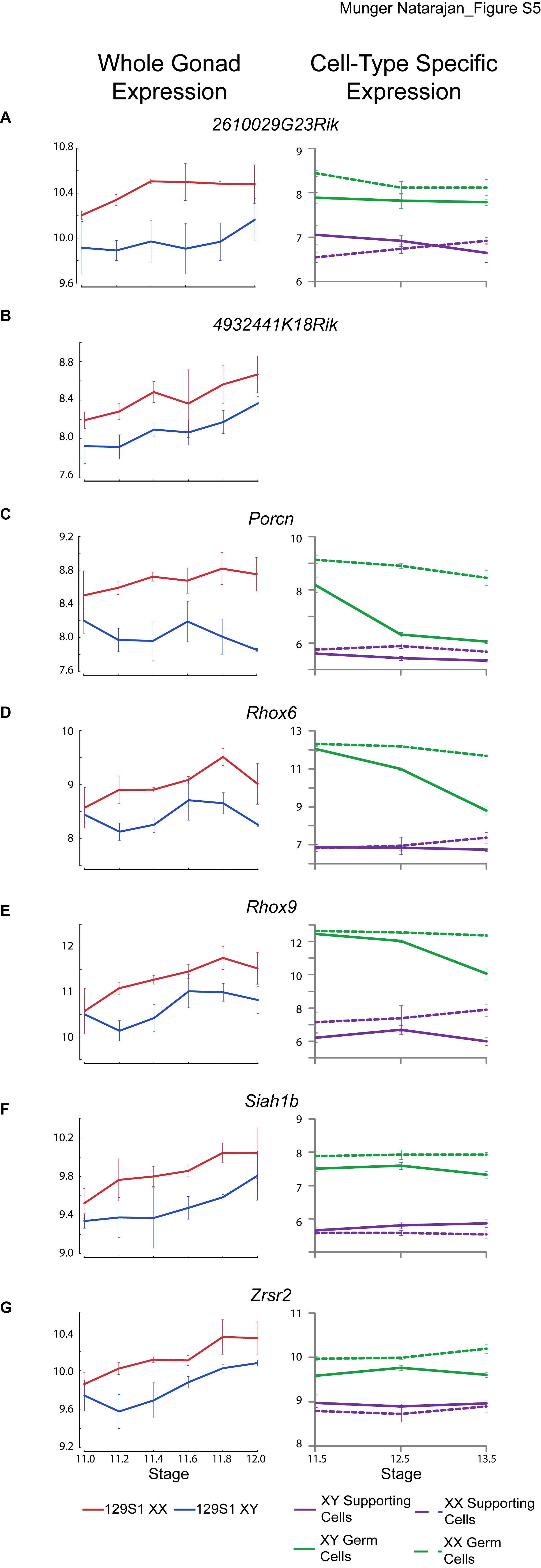

Supplement: Figure S5 — X-linked genes showing higher expression starting at E11.2 are germ-cell enriched. (A–G, left column) 7 genes showing higher expression in XX gonads in 129S1 mice, likely due to the higher number of germ cells in 129S1 mice [S15]. (A–G, right column) Corresponding expression in male (solid line) and female (broken line) in germ cells (green) and supporting cells (purple) from cell-type specific expression data. Note that 4932441K18Rik expression was not captured in the cell-type specific expression dataset. All genes show enriched expression in germ cells. (JPG) [file pgen.1003630.s007.jpg]

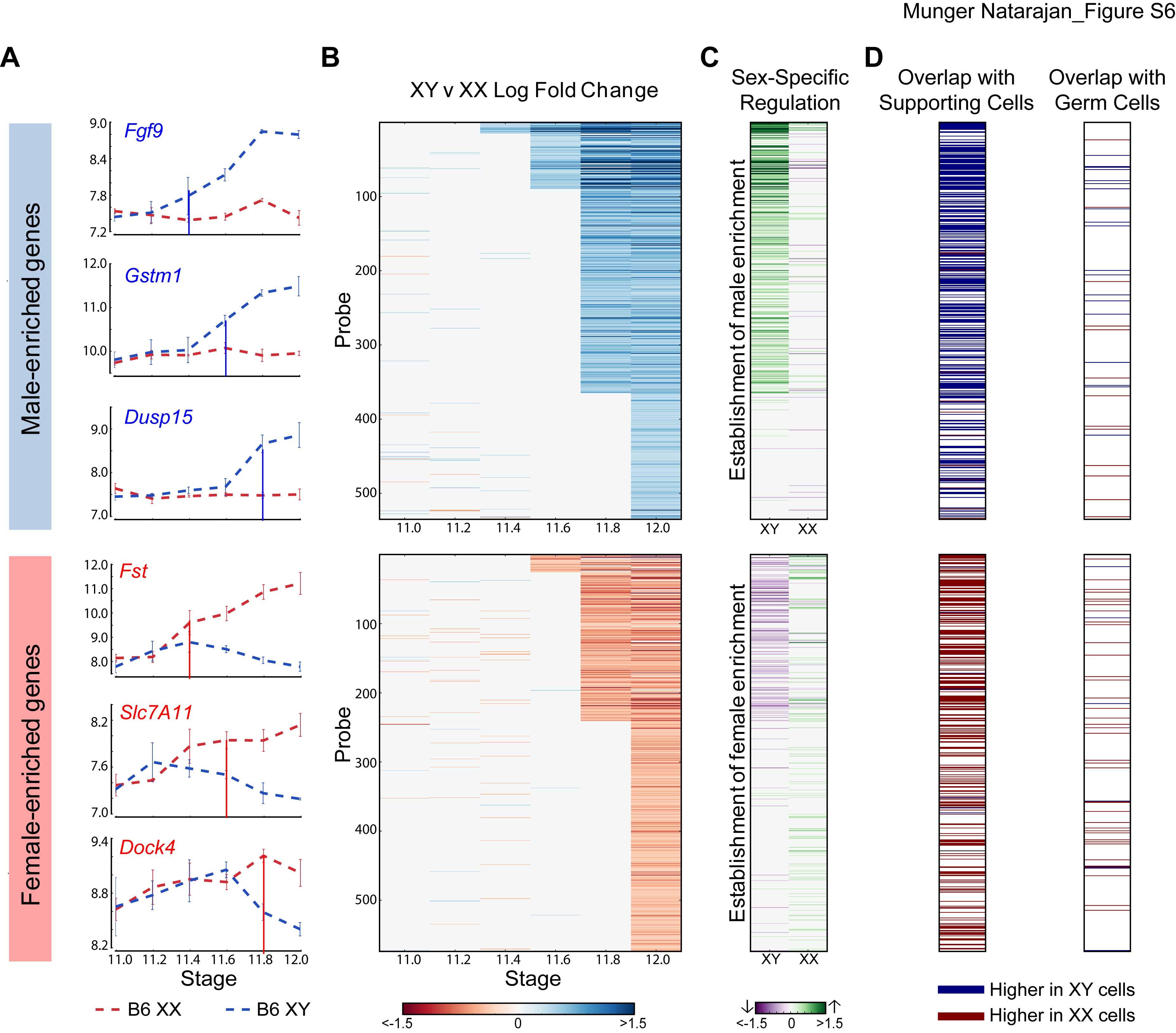

Supplement: Figure S6 — Detailed characterization of dimorphic expression in B6 gonads reveals properties similar to 129S1 gonads. (A) Examples of genes showing higher expression in XY (male-enriched genes, top panel) and XX gonads (female-enriched genes, bottom panel) from B6 mice. Blue and red vertical lines show the time of onset of dimorphic expression. (B) Cascades of dimorphic gene expression identified by the HMM in XY (top panel) and XX gonads (bottom panel). Colors indicate the fold difference between B6 XY and XX gonads at a specific time point. The genes are arranged in order of increasing time of onset of dimorphic expression. (C) Contribution to changes in expression between E12.0 and the time point before the onset of dimorphism are shown for each gene in (B) in XY (column 1) and XX (column 2) gonads. Top panel: male-enriched genes. Bottom panel: female-enriched genes. This analysis shows that male-enriched genes are mostly up-regulated in XY gonads while female-enriched genes are mostly down-regulated in XY gonads. (D) The cascade of genes dimorphically expressed was cross-referenced with cell-type specific expression datasets analyzed at E11.5 and E12.5 [2]. Column 1 shows overlap with genes expressed dimorphically in supporting cells while column 2 shows overlap with genes expressed dimorphically in germ cells. Rows are colored blue or red where the probe was dimorphically expressed and higher in XY cells or higher in XX cells, respectively. As with 129S1 gonads, the highest overlap is seen with the supporting cells for both male- and female-enriched genes in B6 gonads. (JPG) [file pgen.1003630.s008.jpg]

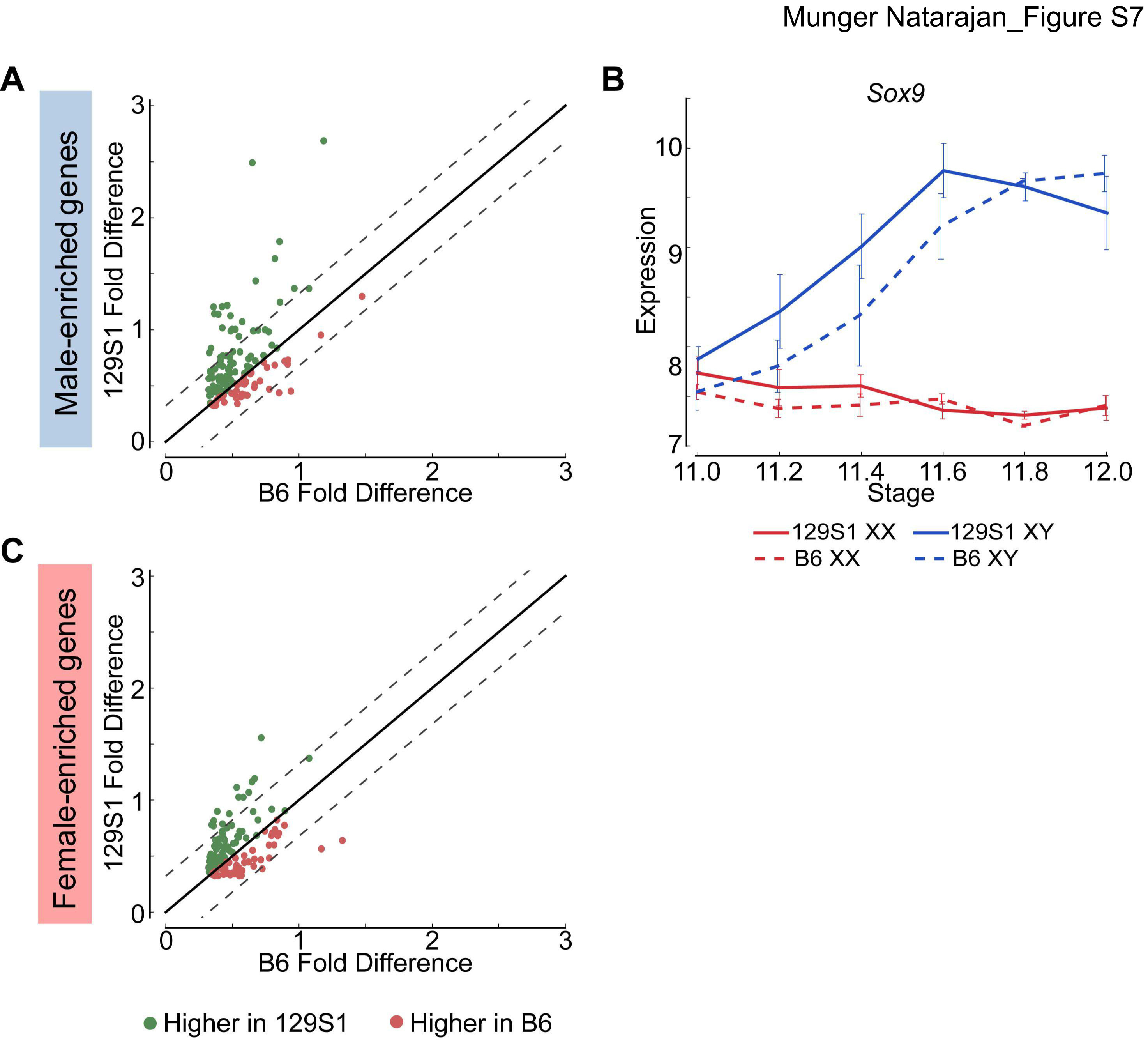

Supplement: Figure S7 — Robust onset of dimorphism in 129S1 mice compared to B6 mice. (A, C) Scatterplot showing XY vs. XX fold difference (A) and XX vs. XY fold difference (C) at the onset of dimorphism for male- and female-enriched genes that are activated at the same stage. Fold difference between 129S1 XY and XX gonads at the onset of dimorphism are plotted on the y-axis and the fold difference between B6 XY and XX gonads at the onset of dimorphism on the x-axis. Onset of dimorphism is more robust in the 129S1 strain for both male- and female-enriched genes. (B) Sox9 becomes dimorphic at E11.2 in 129S1 and B6 gonads. However, the fold difference between XY and XX gonads is higher at E11.2 in 129S1 mice. (JPG) [file pgen.1003630.s009.jpg]

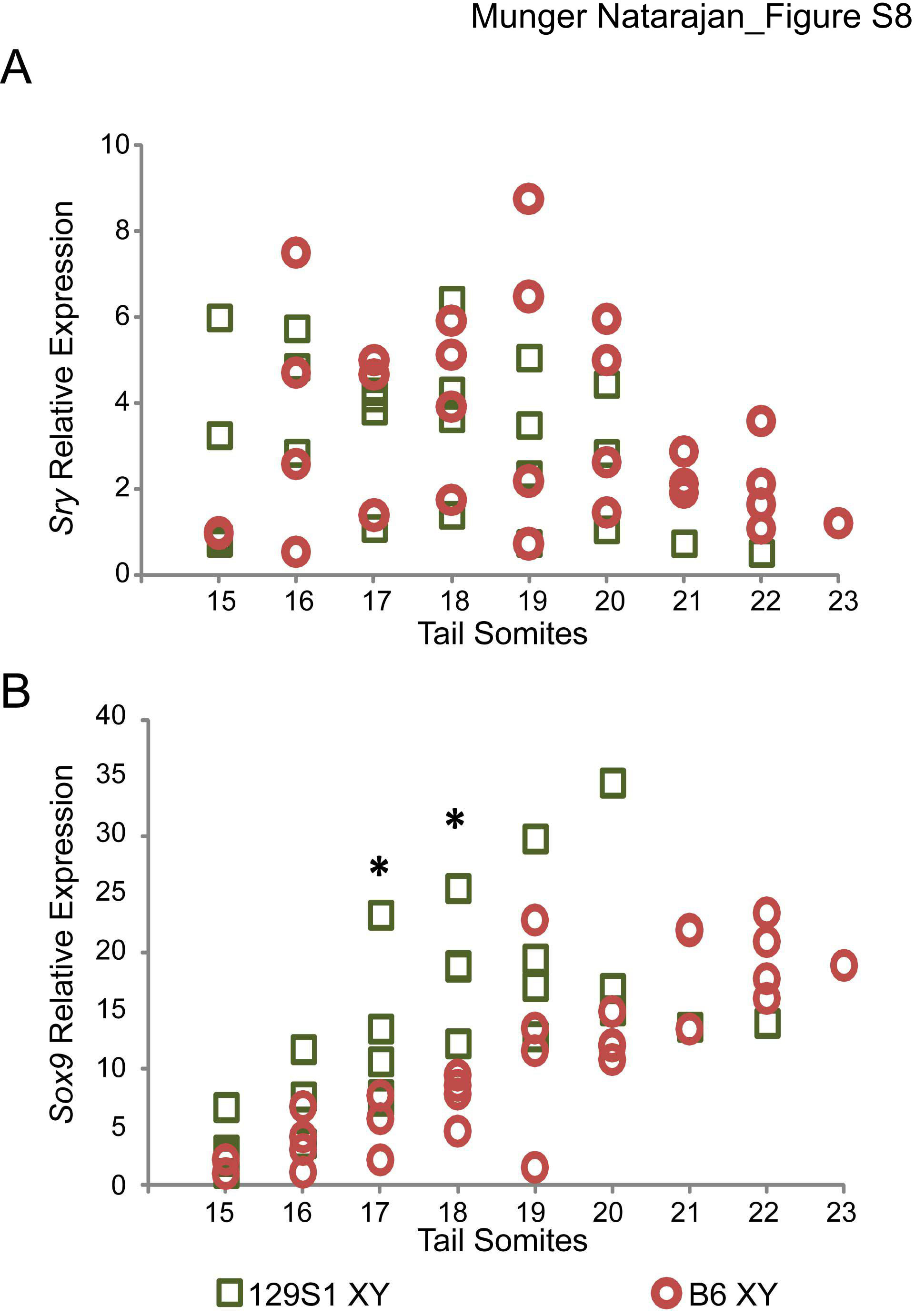

Supplement: Figure S8 — 129S1 and B6 XY gonads show no significant difference in Sry expression but a small difference in Sox9 expression as assayed by qRT-PCR. (A) Sry expression levels are similar in 129S1 and B6 XY gonads between E11.2–E12.0. No statistically significant (p<0.1) differences are detected at any time point in this analysis, however high variability among individuals may mask a small but biologically meaningful strain effect for Sry transcript abundance in this window. (B) Sox9 expression shows significantly different expression (p<0.1 (*) and p<0.05 (**)) at the 17 and 18 tail somite stage. (JPG) [file pgen.1003630.s010.jpg]

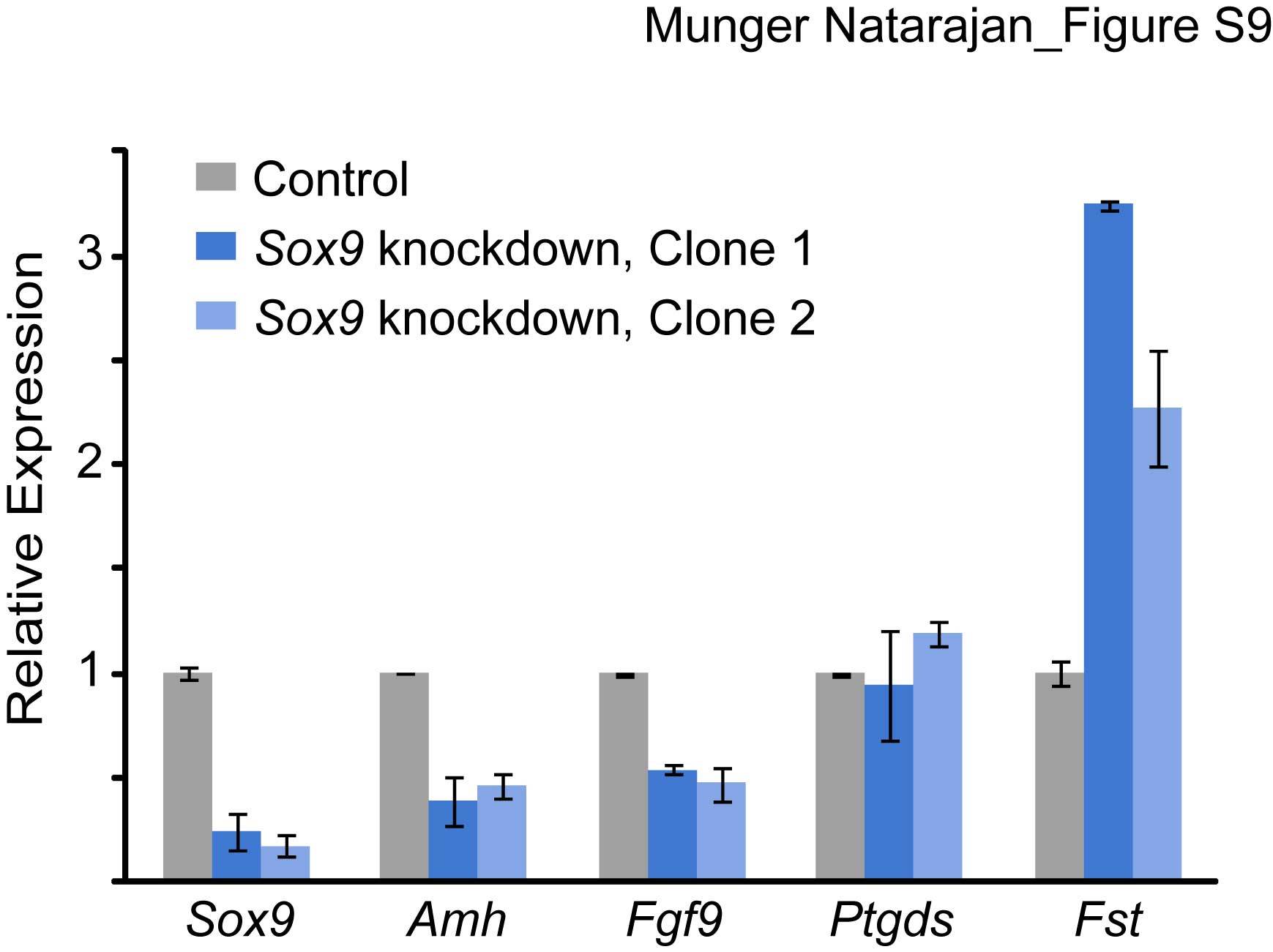

Supplement: Figure S9 — Lentiviral mediated knockdown of Sox9 in gonad primary cell culture results in down-regulation of male-enriched genes. Knockdown of Sox9 resulted in down-regulation of known male-enriched genes such as Amh and Fgf9 and up-regulation of female-enriched gene Fst. However, Ptgds, a known male-enriched gene [35], does not show down-regulation. (JPG) [file pgen.1003630.s011.jpg]

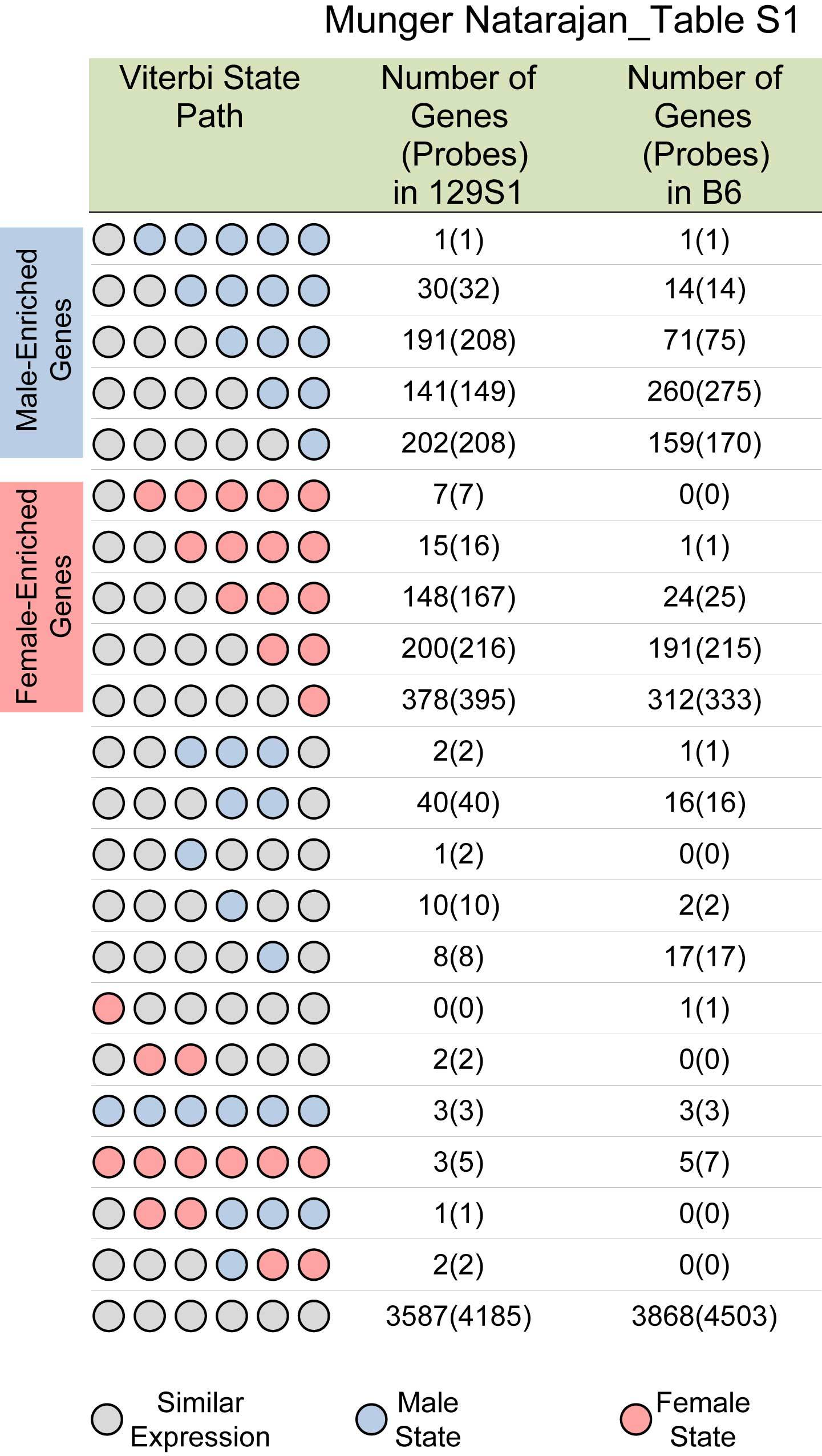

Supplement: Table S1 — Numbers of genes (probes) in each Viterbi state path identified by the HMM for both 129S1 and B6 mice. Each state path has six states, one for each time point, and genes having the same state path are clustered together. Only 3 genes switch from showing higher expression in one sex to higher expression in the other. Most genes that become dimorphic continue showing dimorphism throughout the E11.0–E12.0 window. (JPG) [file pgen.1003630.s012.jpg]

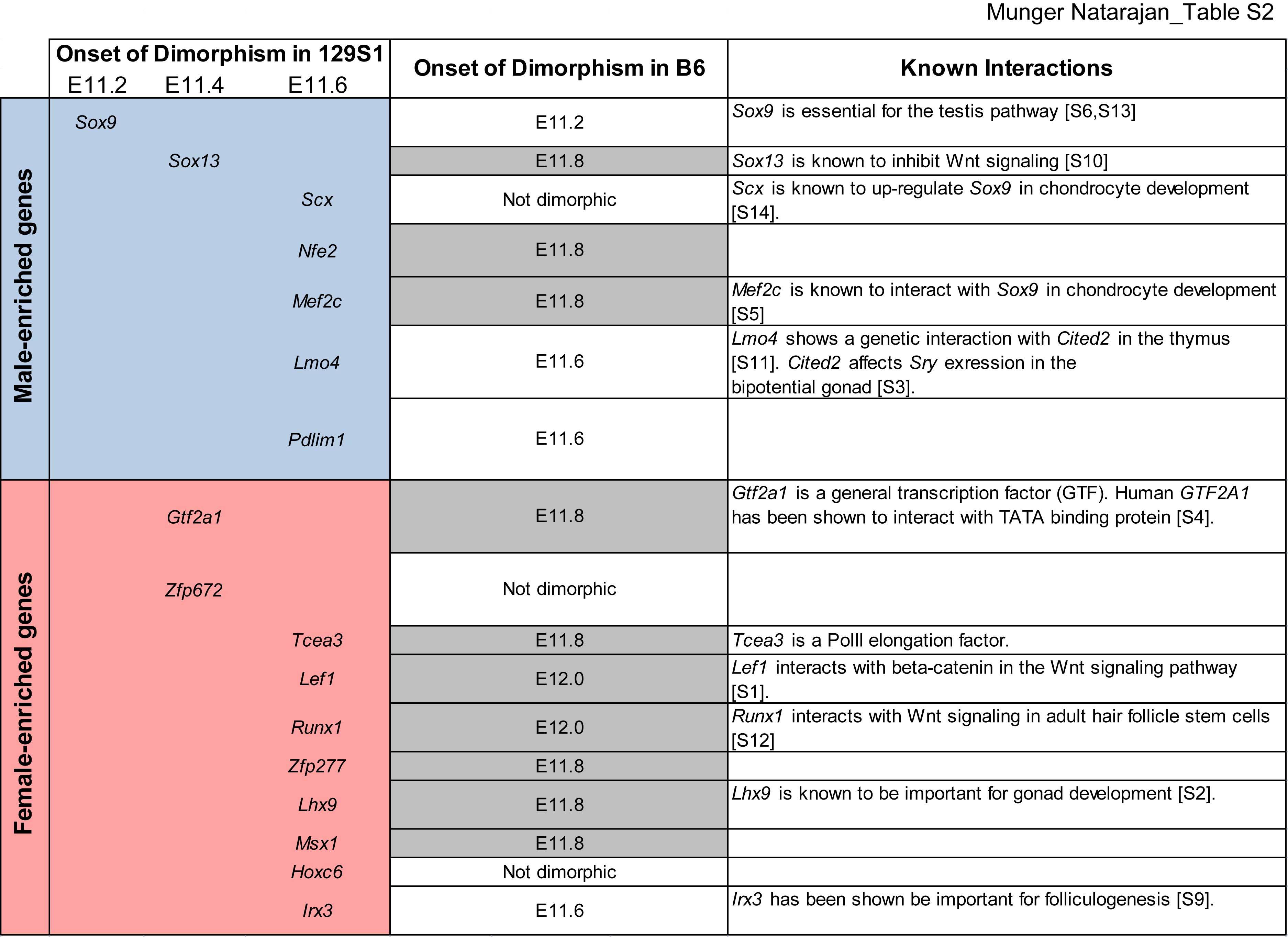

Supplement: Table S2 — Transcription Factors (TFs) and cofactors that are either male- or female- enriched genes and show dimorphic expression by E11.6 in supporting cells in 129S1 mice. Genes showing delayed onset of dimorphism in B6 are shown in grey boxes. Known interactions of the TFs that are relevant to sex determination are also shown. (JPG) [file pgen.1003630.s013.jpg]

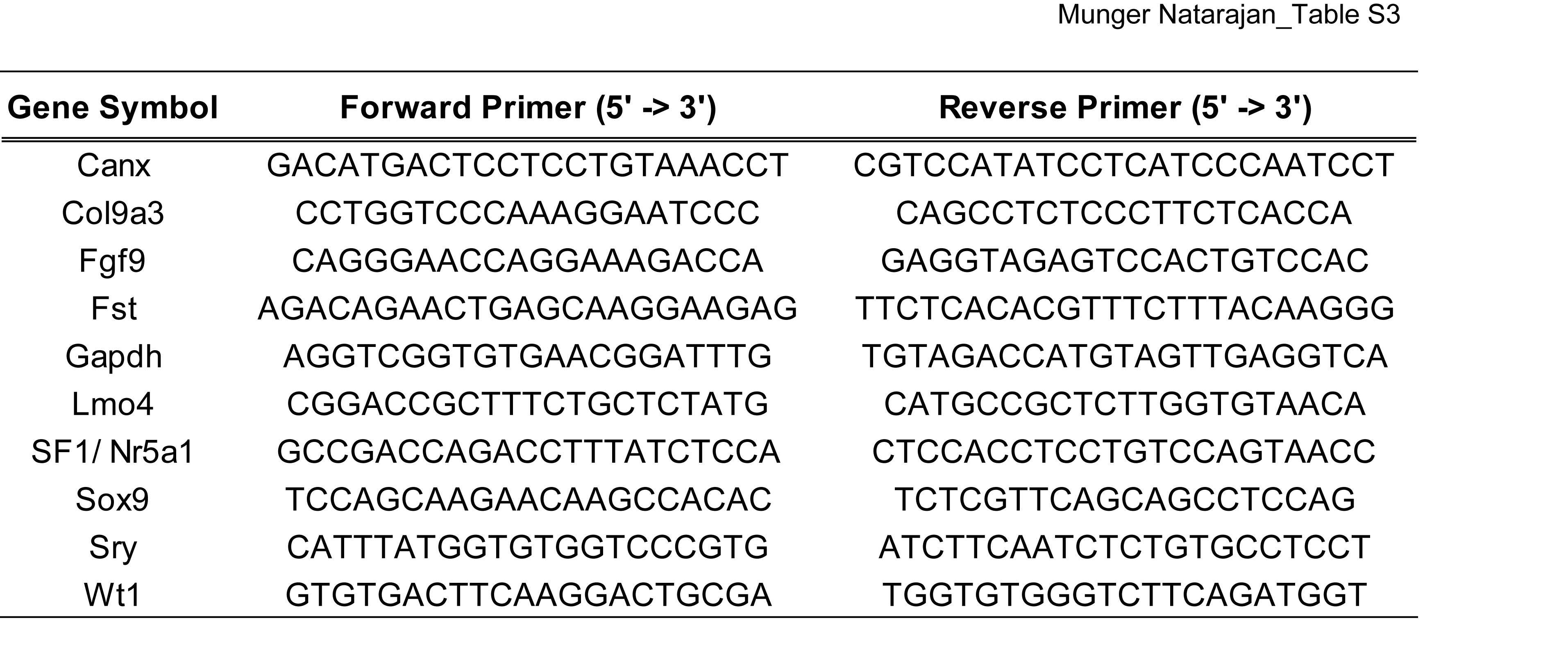

Supplement: Table S3 — List of primers used for qRT-PCR. (JPG) [file pgen.1003630.s014.jpg]
