# Supplementary material for: Fine Time Course Expression Analysis Identifies Cascades of Activation and Repression and Maps a Putative Regulator of Mammalian Sex Determination
Source: PLoS Genet. 2013 Jul 11;9(7):e1003630. doi: 10.1371/journal.pgen.1003630 (PMC3708841; doi:10.1371/journal.pgen.1003630)
Supplement: Text S1 — List of references from Supplemental Material. (DOCX) [file pgen.1003630.s015.docx]

**Text S1. References in Supplemental Materials**

S1. Behrens J, von Kries JP, Kuhl M, Bruhn L, Wedlich D, et al. (1996) Functional interaction of beta-catenin with the transcription factor LEF-1. Nature 382: 638-642.

S2. Birk OS, Casiano DE, Wassif CA, Cogliati T, Zhao L, et al. (2000) The LIM homeobox gene *Lhx9* is essential for mouse gonad formation. Nature 403: 909-913.

S3. Buaas FW, Val P, Swain A (2009) The transcription co-factor CITED2 functions during sex determination and early gonad development. Hum Mol Genet 18: 2989-3001.

S4. DeJong J, Bernstein R, Roeder RG (1995) Human general transcription factor TFIIA: characterization of a cDNA encoding the small subunit and requirement for basal and activated transcription. Proc Natl Acad Sci USA 92: 3313-3317.

S5. Dy P, Wang W, Bhattaram P, Wang Q, Wang L, et al. (2012) Sox9 directs hypertrophic maturation and blocks osteoblast differentiation of growth plate chondrocytes. Dev Cell 22: 597-609.

S6. Foster JW, Dominguez-Steglich MA, Guioli S, Kowk G, Weller PA, et al. (1994) Campomelic dysplasia and autosomal sex reversal caused by mutations in an SRY-related gene. Nature 372: 525-530.

S7. Fujimura N, Vacik T, Machon O, Vlcek C, Scalabrin S, et al. (2007) Wnt-mediated down-regulation of *Sp1* target genes by a transcriptional repressor *Sp5*. J Biol Chem 282: 1225-1237.

S8. Gunther S, Mielcarek M, Kruger M, Braun T (2004) VITO-1 is an essential cofactor of TEF1-dependent muscle-specific gene regulation. Nucleic Acids Res 32: 791-802.

S9. Kim B, Kim Y, Cooke PS, Ruther U, Jorgensen JS (2011) The fused toes locus is essential for somatic-germ cell interactions that foster germ cell maturation in developing gonads in mice. Biol Reprod 84: 1024-1032.

S10. Melichar HJ, Narayan K, Der SD, Hiraoka Y, Gardiol N, et al. (2007) Regulation of gammadelta versus alphabeta T lymphocyte differentiation by the transcription factor SOX13. Science 315: 230-233.

S11. Michell AC, Braganca J, Broadbent C, Joyce B, Franklyn A, et al. (2010) A novel role for transcription factor *Lmo4* in thymus development through genetic interaction with *Cited2*. Dev Dyn 239: 1988-1994.

S12. Osorio KM, Lilja KC, Tumbar T (2011) Runx1 modulates adult hair follicle stem cell emergence and maintenance from distinct embryonic skin compartments. J Cell Biol 193: 235-250.

S13. Wagner T, Wirth J, Meyer J, Zabel B, Held M, et al. (1994) Autosomal sex reversal and campomelic dysplasia are caused by mutations in and around the SRY-related gene SOX9. Cell 79: 1111-1120.

S14. Furumatsu T, Shukunami C, Amemiya-Kudo M, Shimano H, Ozaki T (2010) Scleraxis and E47 cooperatively regulate the Sox9-dependent transcription. Int J Biochem Cell Biol 42: 148-156.

S15. Western PS, Ralli RA, Wakeling SI, Lo C, van den Bergen JA, et al. (2011) Mitotic arrest in teratoma susceptible fetal male germ cells. PLoS One 6: e20736.
